# The Brief Symptom Inventory in the Swiss general population: Presentation of norm scores and predictors of psychological distress

**DOI:** 10.1371/journal.pone.0305192

**Published:** 2024-07-03

**Authors:** Gisela Michel, Julia Baenziger, Jeannette Brodbeck, Luzius Mader, Claudia E. Kuehni, Katharina Roser

**Affiliations:** 1 Faculty of Health Sciences and Medicine,tableniversity of Lucerne, Lucerne, Switzerland; 2 Institute of Psychology, University of Bern, Bern, Switzerland; 3 Institute for Social and Preventive Medicine, University of Bern, Bern, Switzerland; 4 Cancer Registry Bern-Solothurn, University of Bern, Bern, Switzerland; 5 Pediatric Hematology and Oncology, University Children’s Hospital Bern, University of Bern, Bern, Bern, Switzerland; Northwestern University Feinberg School of Medicine, UNITED STATES

## Abstract

Psychological distress is an important and frequent health problem. The Brief Symptom Inventory (BSI) allows screening for psychological distress in clinical, general and research populations. We aimed to provide normative data for the BSI and the BSI-18 for the Swiss general population: We 1) present psychometric properties, 2) develop a Swiss T-standardization and validate it using a clinical sample, 3) describe psychological distress in the Swiss general population and the clinical sample, and 4) compare the means and T-standardized scores of the Swiss general population to different German norm populations. Using a cross-sectional study design, we invited a representative sample of the Swiss general population aged 18–75 years to the study. A sample of psychotherapy outpatients had competed the BSI before start of their therapy. We calculated scores for the nine scales of the BSI (three of them constitute the BSI-18), the T-standardization and the following BSI indices: Global Severity Index (*GSI*), Positive Symptom Total (PST), Positive Symptom Distress Index (PSDI), and Caseness (reaching T≥63 on the *GSI* or T≥63 on at least two of the scales). A total of 1238 general population participants completed the BSI (41.8% male; mean age 48.9 years). The BSI had good psychometric properties. The Swiss T-standardization showed good validity when applied in the clinical sample. Females reached a significantly higher *GSI* score than males (p<0.001). Older participants (p = 0.026), those with higher education (p <0.001), and those employed or retired (p<0.001) reached lower scores than participants aged 18–25 years, those with compulsory schooling, and unemployed participants, respectively. A total of 18.1% (CI: 16.0–20.5) participants of the general population and 75.2% (CI: 73.7–76.7) of the psychotherapy patients were considered cases with psychological distress. Our study presents detailed normative data for the BSI and the BSI-18 based on a representative sample of the Swiss general population. This information will be helpful for clinical applications and research in the Swiss and international context.

## Introduction

Psychological distress can be described as a set of painful mental and physical symptoms [1] and is an important and frequent problem. The prevalence of mental health problems in general populations was estimated at around 12% globally, with depressive disorders contributing most to mental disorder related disability adjusted life years (DALYs) [2]. In Switzerland, a representative study found around 15% of the population reporting psychological distress [3]. A significant proportion of the population reports symptoms of depression or anxiety [4, 5], with recent data showing that 12% of the Swiss population reported medium to severe symptoms of depression and 9% symptoms of anxiety disorder [6]. The prevalence, especially of depression, has increased during the COVID-19 pandemic [7]. Mental health disorders and symptoms of psychological distress are more frequent in subgroups of the population such as individuals living in difficult social conditions (e.g. unemployment, migrant status), certain age groups (especially in young and old people), or with specific diseases (e.g. cancer patients or survivors of cancer) [2, 8–11]. The costs of depression only were estimated at 8 billion Euro annually in Switzerland, with around half of the costs being due to workplace absence [12].

Psychological distress is an important patient reported outcome (PRO) [13, 14], and frequently used as predictor or covariate in research on various health-related outcomes [15–17]. Therefore, it is important to have a valid and reliable assessment tool, which allows to screen for psychological distress, and which can provide information on normative comparison populations for national and international studies.

The Brief Symptom Inventory (BSI) has been developed by Derogatis in the early 1990s [18] based on the more extensive Symptom Check List-90 [19]. It has been translated in many languages and normative data are available for many countries and subpopulations [20–30]. The BSI consists of 53 items, which have to be answered on a 5-point scale ranging from 0 (not at all) to 4 (extremely) relating to the past 7 days. Items can then be summarized to 9 symptom scales: *Somatization*, *Obsession-Compulsion*, *Interpersonal Sensitivity*, *Depression*, *Anxiety*, *Hostility*, *Phobic Anxiety*, *Paranoid Ideation*, *Psychoticism*. In addition, three global indices (summary scores) can be calculated: *Global Severity Index* (*GSI*, indicating the global distress level), *Positive Symptom Total* (*PST*, indicating the number of symptoms an individual reports) and *Positive Symptom Distress Index* (*PSDI*, indicating the average distress level). The BSI further allows calculating a *Case* indicator suggestive of a person experiencing clinically relevant levels of psychological distress. More recently, a shorter version of the BSI was developed, the BSI-18, including 18 items of the BSI and resulting in 3 symptom scales (*Somatization*, *Depression* and *Anxiety*) and a GSI *(GSI-18)* [31]. The *Somatization* scale of the BSI-18 has only six items, unlike the original scale with seven items; the other two scales remain the same. The BSI scores are often presented as T-Scores with a T = 50 (SD = 10) representing the mean in the population, i.e. 50% of the norm population scored below and 50% above this score.

The *Case* indicator can be helpful to indicate a possibly high level of subjective burden with a need for treatment. It has been used in many publications to indicate the prevalence of psychological distress in various populations (some of them using the BSI, some using the BSI-18). Often, the original definition by Derogatis [18] had been used defining a *case* as T≥63 on the *GSI* or T≥63 on at least two of the scales: e.g. in patients with traumatic brain injury [32, 33], earthquake survivors [34], survivors of hematopoietic stem cell transplant (HSCT) [35], cancer patients [36] or drug-dependent adults [37]. In some studies, especially in cancer patients, alternative case rules for the BSI-18 have been used such as T = 50 [36, 38, 39] or T = 57 [40]as cut-off. Franke and colleagues [41] recently presented a new routine for the coding of *caseness* using a sample of orthopaedic patients: “no” distress: if a maximum of one scale has a T≥60; “mild” psychological distress: if ‘T on two scales’ and/or ‘T on GSI’ is ≥60 and <63; “remarkable” psychological distress: if ‘T on two scales’ and/or ‘T on GSI’ is ≥63 and <70; “severe” psychological distress: if ‘T on two scales’ and/or ‘T on GSI’ is ≥70. They highlight the relevance of these additional categories by their importance to inform prevention and intervention. For individuals with mild distress, a short diagnostic interview and if necessary some additional tests might be recommended, as well as re-testing after a relatively short time. For those with remarkable distress, interviews and additional tests are recommended. Interventions might be needed, as well as close follow-up. For the group with severe psychological distress, in-depth exploration and intervention should be available.

To date, Swiss normative data are lacking and Swiss studies had to rely on normative data from other countries, often from Germany [20, 29, 30]. However, it is not clear if the German norm populations adequately represent the Swiss general population, and can thus be used as an appropriate comparison in the clinical and research context. Apart from cultural differences, two of the three official languages of Switzerland (French and Italian) are not represented in normative data from Germany.

Our objective was to provide normative data for the BSI and the BSI-18 for the Swiss general population. Using a representative sample of the Swiss general population, we aimed to: 1) present psychometric properties of the BSI for Switzerland, 2) develop Swiss T-standardization for the BSI (nine scales and three global indices) and validate it using a clinical sample of psychotherapy outpatients, and 3) describe psychological distress in the Swiss general population, subgroups with different characteristics, and the clinical sample. In addition to the original BSI, we also present these results for the BSI-18. 4) Finally, we compared the means and T-standardized scores (according to the German standardizations) of the Swiss general population to different German norm populations.

## Methods

### Ethics approval

Ethical approval was granted by the Ethics Committee of Northwest and Central Switzerland (EKNZ 2015–075; 26 March 2015), and participants provided written informed consent. The reporting of the study is in accordance with the STROBE guideline (S1 Checklist).

### Participants and procedure

#### Sample of Swiss general population

The representative sample for this cross-sectional study was provided by the Swiss Federal Statistical Office (SFSO) and consisted of 3000 households, which were randomly selected according to the distributions of age, sex, and language in Switzerland (for the sample distribution see Table 1: ‘weighted’ column; for further details on characteristics of the Swiss general population see [42]).The total sample included 7052 Swiss residents. For the study, we only included adults aged 18–75 years at sample selection in 2015, leaving 5644 persons eligible for the study.

**Table 1:** Characteristics of respondents who completed the Brief Symptom Inventory (BSI) (n = 1238).

	Unweighted		Weighted
	N	%	%
**Sex (from SFSO)**			
Male	517	41.8	48.2
Female	721	58.2	51.8
**Age [years]**			
18–25	92	7.4	9.2
26–35	168	13.6	17.4
36–45	231	18.7	18.9
46–55	287	23.2	22.4
56–65	237	19.1	17.4
66–75	223	18.0	14.9
**Language of questionnaire**			
German	910	73.5	72.4
French	260	21.0	22.0
Italian	68	5.5	5.6
**Nationality (from SFSO)**			
Swiss	1077	87.0	77.6
Other	161	13.0	22.4
**Migration background**			
No migration background	970	78.4	70.3
Migration background	268	21.7	29.7
**Education**			
Compulsory schooling	96	8.2	8.2
Vocational training	563	48.3	46.5
Upper secondary education	294	25.2	24.3
University education	213	18.3	21.0
**Employment**			
Unemployed	148	12.3	13.0
Employed	835	69.2	71.7
Retired	223	18.5	15.3
	**Mean**	**SD**	**Mean**
Age	48.9	15.2	46.8
	**Min**	**Max**	**CI**
	17.9	76.3	45.9–47.7

Participants were weighted according to the distribution of sex, age, and nationality in all eligible persons (n = 5644)

Abbreviations: SFSO: Swiss Federal Statistical Office, SD: Standard deviation, Min: minimum, Max: maximum, CI: 95% confidence interval

University education: minimum of an education equivalent to a master according to bologna system

Eligible persons were individually contacted by the study team with an information letter in the language indicated by the SFSO (German, French, or Italian) between May 2015 and June 2016. Approximately two weeks later, we sent a questionnaire with a cover letter and a pre-paid return envelope. Non-respondents received a reminder letter with an additional copy of the questionnaire and a pre-paid return envelope approximately five weeks after the initial questionnaire. Participants provided informed consent to participate in the study.

#### Clinical sample of psychotherapy outpatients

We received the BSI data from a sample of 3152 individuals who were >18 years and sought treatment at the outpatient clinic of the Institute of Psychology of the University of Berne, Switzerland, the Klaus-Grawe-Institute for Psychological Therapy Zurich, Switzerland, or an independent psychotherapist trained at one of these institutions between 2000 and 2012 [43]. No identifying information was available for this sample. For a subsample of psychotherapy patients, a clinical diagnosis was available (depression, social phobia, agoraphobia / panic disorder, generalized anxiety disorder, phobia, somatization, compulsory disorder, psychosis).

### Measurements

#### Brief Symptom Inventory

Participants from both samples completed the BSI in a paper-based questionnaire. We calculated the following indices: **Sum scores**: Sum scores for each scale were calculated by summing up the items of the respective scale, and all items for the *GSI*. If no more than one item was missing on a scale, the item was replaced by the rounded mean of the remaining items of the respective scale. For the *GSI*, the rounded mean was included for up to 12 missing items, as instructed by the BSI manual [20]. If a participant had more than one item missing on a scale or 12 items for the *GSI*, the respective scale or the *GSI* was not calculated. In addition to the nine original BSI scales and the *GSI*, we also calculated the scores for the respective scales and the *GSI-18* of the BSI-18 including the items of the following scales: *Somatization-6 items*, *Depression* and *Anxiety*. **Mean scores:** The mean of all available items of the respective scale was calculated for all scales and the *GSI* (only if no more than one item per scale or 12 items for the *GSI* were missing). **Positive Symptom Total (PST)**: The PST was calculated by summing up the number of items with non-zero responses (separately for each scale, and for all items). The PST reveals the number of symptoms as reported by the respondent. **Positive Symptom Distress Index (PSDI):** The PSDI is the *GSI*-sum-score divided by the PST of all items. This index provides information about the intensity of distress experienced by the respondent [20]. **T-scores**: The T-standardization was based on the sum score of each scale. We used the T-standardization as presented by Franke to obtain German T scores [20]. After developing the T-standardization for Switzerland, we calculated Swiss T-Scores. From the T-Scores a **scale-caseness** index was calculated for each scale and the *GSI*. Scale-caseness was defined as T≥63. Finally, individuals were considered a **case with psychological distress** if they reached T≥63 on the *GSI* or T≥63 on at least two of the scales [20].

#### Socio-demographic information

From the SFSO we received the following information: sex, age in years (categorized into 10-year age groups: 18–25, 26–35, 36–45, 46–55, 56–65, 66–75 years), language region (according to questionnaire completed: German, French, Italian), nationality (Swiss, other), marital status (single, married, divorced/widowed).

In the questionnaire we assessed: education, employment, migration background. Detailed categories are presented in Table 1. For the clinical sample, only information on sex was available.

### Statistical analyses

We used sample weighting to account for differences between study participants and non-participants of the Swiss general population. To obtain a representative sample of the Swiss general population, participants were weighted according to the distribution of sex, age, and nationality in all eligible persons (n = 5644). All analyses were done using STATA 15.1 and 18.0.

1) To present psychometric properties of the BSI for Switzerland, we calculated the number of missing items per scale (before imputation), the number of participants reporting the minimum (floor) or the maximum (ceiling) score on each of the scales (all from unweighted sample) and Cronbach’s Alpha (from weighted sample). In addition, we present item-scale correlations, item-rest correlations, average inter-item covariances, Cronbach’s alpha without the item for each scale and inter-scale correlations. We performed a confirmatory factor analysis (CFA) using the SEM command and maximum likelihood estimation in STATA 18.0.

2) To develop and present Swiss T-standardization for the BSI scales and the *GSI*, we calculated the standardized area T-Score: We calculated the cumulative percentile of each sum score for each of the scales using the weighted sample. The mean cumulative percentile of two consecutive scores was used for the area transformation into T-Scores [44]. Similar to the German T-Norms [20], we decided to have a maximum T-Score of 80 (indicating >99 cumulative percentile). To validate the Swiss T-scores, we used the clinical sample of psychotherapy outpatients. We calculated the same overall scores: sum scores, mean scores, PST, PSDI, T-Scores and Case-index (the proportion of patients reaching a critical Score of T≥63). We used t-tests to compare the T-score of the patient group with the mean T = 50 in the norm population. To evaluate the construct validity, we calculated proportion of *Cases* in patients with a specific diagnosis and compared it with the group of patients without the specific diagnosis using chi^2^ test for proportions.

3) To describe psychological distress in the Swiss general population, for subgroups with different characteristics (by sex, age group, language region, education, employment, and migration background), and for the clinical sample, we calculated the means and 95% confidence intervals (CI) for the following scores: sum scores, mean scores, PST, PSDI and T-Scores (using the German and Swiss standardization separately). We used univariable linear regression analyses on the weighted sample to analyze differences in BSI sum scores by sex, age group, language region, education, employment, and migration background. In multivariable regression analyses on the weighted sample we included the variables, which were significantly associated at p<0.05 in the univariable analysis of the respective scale. In addition, we calculated the proportion of *cases* with psychological distress in the total population and different subgroups (sex, age group, language region, education, employment, and migration background). Differences in the proportion of *cases* between subgroups were calculated using univariable logistic regression analyses (using the weighted sample).

4) Finally, to compare the scores of the Swiss general population with scores of different German norm populations we used t-tests and compared means and T-scores of the Swiss General population (based on three different German standardizations[20, 29, 30]) to the different German norms.

## Results

Of 5644 Swiss residents contacted, 1255 returned a completed questionnaire (response rate 22.2%; male participants n = 524, 41.8%; mean age 49.0 years, SD = 15.2; S1 Table in (S1 Appendix)). Respondents were more likely to be female (<0.001), older (<0.001), of Swiss nationality (<0.001), and being married or divorced/widowed (p = 0.003) compared to non-responders. The clinical sample consisted of 3152 outpatients. For 761 we had information on sex with 430 female participants (56.5%). For 1005 (31.9%) outpatients we had information on their diagnosis (S2 Table in (S1 Appendix)).

### Aim 1: Test statistics and reliability

A total of 1238 persons of the Swiss general population sample completed enough items of the BSI to be included in the remaining analyses (Table 1). Most participants completed the full BSI without any missing items (n = 1149, 92.8%, Table 2). All scales were highly skewed such that a large number of participants reported no symptom on the respective scale (Table 2). This was most predominant on *Phobic Anxiety*, where 790 (63.8%) of participants reported no symptom. The reliability of the scales was good (Cronbach’s α between 0.669 for *Somatization 6 items* and 0.954 for *GSI;* Table 2). Information on item-scale correlations, item-rest correlations, average inter-item correlations, Cronbach’s alpha without the item for each item is available in online S3 Table in (S1 Appendix). The inter-scale correlation was relatively high ranging from 0.421 (*Somatization* and *Paranoid Ideation*) to 0.767 (*Depression* and *Psychoticism*, Table 3). The correlation with the *GSI* was highest for *Interpersonal Sensitivity* (r = 0.851). Goodness of fit indices for the CFA showed relatively mediocre model fit (details are available in online S4 Table in S1 Appendix).

**Table 2:** Psychometric properties of the BSI in the Swiss general population.

	Number of items in the scale:	Number of missing items					Number of participants reaching the min (Floor) and the max (Ceiling) on each of the scales							
		none	1 missing (1–12 for GSI)		>1 (>12 for GSI)		Sum scores		T-Scores (German norms)		T-Scores (Swiss norms)		Average inter-item correlation	Cronbach’s alpha
		N	N	%	N	%	Floor	Ceiling	Floor	Ceiling	Floor	Ceiling		
Somatization	7	1224	13	1.0	18	1.4	437	1	437	8	437	3	0.258	0.707
Obsessive-compulsive tendencies	6	1213	22	1.8	20	1.6	246	1	246	12	246	1	0.409	0.803
Interpersonal sensitivity	4	1234	5	0.4	16	1.3	489	1	489	18	489	2	0.507	0.797
Depression	6	1225	12	1.0	18	1.4	546	2	546	7	546	2	0.436	0.817
Anxiety	6	1225	12	1.0	18	1.4	405	1	405	6	405	2	0.397	0.775
Hostility	5	1225	12	1.0	18	1.4	342	1	342	8	342	2	0.338	0.698
Phobic anxiety	5	1233	13	1.0	9	0.7	790	2	790	15	790	2	0.381	0.715
Paranoid ideation	5	1231	6	0.5	18	1.4	394	1	394	5	394	2	0.396	0.763
Psychoticism	5	1220	17	1.4	18	1.4	664	1	664	12	664	2	0.303	0.679
GSI (accepted number of missing items ≤12)	53	1149	89	7.1	17	1.4	46	1	46	33	46	3	0.284	0.954
**BSI-18**														
Somatization (6 items)	6	1227	10	0.8	18	1.4	505	2			437	8	0.254	0.669
GSI-18 (accepted number of missing items ≤2)	18	1206	30	2.4	19	1.5	184	1			184	2	0.287	0.877

Abbreviations: BSI Brief Symptom Inventory, BSI-18 Brief Symptom Inventory 18, GSI Global Severity Index, GSI-18 Global Severity Index for the Brief Symptom Inventory 18 (only including items of the Somatization (6 items), Depression and Anxiety scale, min minimum, max maximum

**Table 3:** Inter-scale correlations for the 9 scales and the Global Severity Index (GSI) of the BSI and the BSI-18 (Pearson correlation from weighted sample).

	1	2	3	4	5	6	7	8	9	10	11	12
1 Somatization	1.000											
2 Obsessive-compulsive tendencies	0.540	1.000										
3 Interpersonal sensitivity	0.434	0.657	1.000									
4 Depression	0.444	0.675	0.730	1.000								
5 Anxiety	0.569	0.671	0.679	0.641	1.000							
6 Hostility	0.447	0.584	0.640	0.587	0.639	1.000						
7 Phobic anxiety	0.467	0.514	0.599	0.578	0.595	0.448	1.000					
8 Paranoid ideation	0.421	0.524	0.692	0.568	0.577	0.591	0.520	1.000				
9 Psychoticism	0.461	0.666	0.711	0.767	0.659	0.602	0.601	0.634	1.000			
10 GSI	0.678	0.830	0.851	0.843	0.843	0.765	0.721	0.763	0.842	1.000		
**BSI-18**												
11 Somatization (6 items)	-	-	-	0.450	0.566	-	-	-	-	-	-	
12 GSI-18	-	-	-	0.857	0.885	-	-	-	-	-	0.766	1.000

Abbreviations: BSI Brief Symptom Inventory, BSI-18 Brief Symptom Inventory 18, GSI Global Severity Index, GSI-18 Global Severity Index for the Brief Symptom Inventory 18 (only including items of the Somatization (6 items), Depression and Anxiety scale)

### Aim 2: Swiss T-standardization

Descriptive information on the Swiss T-standardization scores is presented in Table 4, and details on the standardization are presented in S7-S9 Tables in (S2 Appendix). By definition the mean T-score equals T = 50 (with a standard deviation of 10). Due to the limited number of values in the sum-score of each scale used for standardization, the calculated standardized mean might be slightly higher or lower than T = 50.

**Table 4:** Descriptive statistics for the scales and the GSI of the BSI for the representative Swiss population sample (Sum score, Mean score, T-Scores (standardization according to German and Swiss norms), Positive Symptom Total; all based on weighted analyses).

	Sum scores			Mean scores			BSI Positive Symptom Total (PST)			T- Standardization (German norms)			T- Standardization (Swiss norms)		
	Mean	95% CI		Mean	95% CI		Mean	95% CI		Mean	95% CI		Mean	95% CI	
Somatization	1.97	1.82	2.12	0.28	0.26	0.30	1.57	1.47	1.66	50.2	49.7	50.8	50.4	49.9	50.9
Obsessive-compulsive tendencies	3.12	2.93	3.31	0.52	0.49	0.55	2.46	2.35	2.57	49.6	49.0	50.2	50.1	49.6	50.7
Interpersonal sensitivity	1.71	1.57	1.84	0.43	0.39	0.46	1.28	1.20	1.36	49.7	49.1	50.3	50.5	50.0	51.0
Depression	1.94	1.76	2.12	0.32	0.29	0.35	1.45	1.35	1.54	50.5	49.9	51.1	50.5	49.9	51.0
Anxiety	2.15	1.99	2.31	0.36	0.33	0.38	1.60	1.51	1.69	50.1	49.5	50.7	50.5	49.9	51.0
Hostility	1.83	1.70	1.95	0.37	0.34	0.39	1.37	1.30	1.45	50.4	49.8	51.0	50.1	49.6	50.6
Phobic anxiety	0.85	0.74	0.95	0.17	0.15	0.19	0.62	0.56	0.68	50.6	50.1	51.1	50.4	50.0	50.9
Paranoid ideation	2.25	2.09	2.41	0.45	0.42	0.48	1.71	1.62	1.80	52.3	51.7	52.9	50.4	49.9	51.0
Psychoticism	1.18	1.06	1.30	0.24	0.21	0.26	0.88	0.81	0.95	51.5	51.0	52.1	50.6	50.2	51.1
GSI	18.57	17.44	19.70	0.35	0.33	0.37	14.14	13.54	14.73	49.8	49.0	50.5	50.0	49.5	50.6
**Positive Symptom Distress Index (PSDI)**							1.24	1.22	1.26						
**BSI-18**															
Somatization (6 items)	1.63	1.50	1.75	0.27	0.25	0.29							51.8	51.3	52.4
GSI-18	5.71	5.32	6.10	0.32	0.30	0.34							50.2	49.6	50.7

Abbreviations: BSI Brief Symptom Inventory, BSI-18 Brief Symptom Inventory 18, GSI Global Severity Index, GSI-18 Global Severity Index for the Brief Symptom Inventory 18 (only including items of the Somatization (6 items), Depression and Anxiety scale

Sum Score: sum of all items of respective scale; Mean score: mean of all items of respective scale

T-standardized scores of the clinical sample (standardization according to the Swiss norms) showed that psychotherapy outpatients reached significantly higher T-scores on all scales and the *GSI* as compared to the Swiss general population (Table 5). On all scales, a considerable number of patients reached the critical score of T≥63 (from 41.9% on *phobic anxiety* to 64.2 on the *GSI*), and 75.2% (CI: 73.7–76.7) of patients were considered overall *cases* (T≥63 on at least two scales or T≥63 on the *GSI*). Scores for the clinical sample are presented in Table 5. Overall, construct validity was adequate (S6 Table in (S1 Appendix))

**Table 5:** Descriptive statistics for the scales and the GSI of the BSI for a clinical sample of psychotherapy outpatients (Sum score, Mean score, T-Scores (Standardization according to German and Swiss norms), Positive Symptom Total; All based on weighted analyses.

		Sum score		Means score		BSI Positive Symptom Total (PST)		T- Standardization (German norms)		T- Standardization (Swiss norms)		Difference to Swiss norm of T = 50	Cases with T≥63
	N	Mean	SD	Mean	SD	Mean	SD	Mean	SD	Mean	SD	p^a^	%
Somatization	3152	5.97	5.76	0.85	0.82	3.12	2.30	60.66	12.98	60.04	12.05	<0.001	43.0
Obsessive-compulsive tendencies	3152	8.39	5.54	1.40	0.92	3.99	1.71	63.43	12.31	61.98	10.48	<0.001	57.6
Interpersonal sensitivity	3151	5.48	3.88	1.37	0.97	2.61	1.30	64.34	12.16	62.14	9.43	<0.001	53.1
Depression	3152	8.25	6.05	1.38	1.01	3.84	1.87	66.53	11.40	64.28	9.99	<0.001	59.9
Anxiety	3151	7.19	5.39	1.20	0.90	3.46	1.77	65.28	12.37	63.03	10.21	<0.001	53.3
Hostility	3152	4.98	3.93	1.00	0.79	2.41	1.36	62.26	11.82	60.79	10.85	<0.001	46.3
Phobic anxiety	3152	3.84	4.43	0.77	0.89	1.88	1.68	62.15	13.27	56.75	15.34	<0.001	41.9
Paranoid ideation	3152	4.95	4.26	0.99	0.85	2.63	1.66	60.80	11.92	57.99	10.76	<0.001	37.5
Psychoticism	3152	4.80	4.17	0.96	0.83	2.30	1.49	65.04	11.91	62.29	10.41	<0.001	53.3
GSI	3150	58.34	37.05	1.10	0.70	28.46	12.09	68.32	11.87	64.38	9.51	<0.001	64.2
2 scales T≥63 or GSI T≥63													75.2
**Positive Symptom Distress Index (PSDI)**						1.89	0.60						
**BSI-18**													
Somatization (6 items)	3152	5.00	4.95	0.83	0.82					61.76	12.35	<0.001	49.4
GSI-18 (accepted number of missings ≤2)	3151	20.45	13.92	1.14	0.77					64.86	9.96	<0.001	

a p from t-test (comparison with the Swiss norm score based on the Swiss general population sample).

Sum Score: sum of all items of respective scale; Mean score: mean of all items of respective scale

### Aim 3: Psychological distress in the Swiss general population, in subgroups with different characteristics, and a sample of psychotherapy patients

Descriptive information on psychological distress for the representative Swiss population sample are presented in Table 4 (details for subgroups according to sex, age, language region, education, employment, and migration background are presented in S10 Tables a)-g) in (S3 Appendix)). Univariable regression analyses showed that females reached a significantly higher *GSI* score than males (p<0.001). Older participants (overall p = 0.026), those with higher education (overall p <0.001), and those employed or retired (overall p<0.001) reached lower scores than participants aged 18–25 years, those with compulsory schooling, and unemployed participants, respectively (Table 6). These associations remained significant in the multivariable regression analysis. Detailed analyses for each of the scales are presented in S11 Table (univariable regression analyses) and supplemental S12 Table (multivariable regression analyses) in (S4 Appendix). Regarding the different scales (multivariable regressions), females reached higher sum scores than males in *Somatization (*also *Somatization 6-items)*, *Interpersonal Sensitivity*, *Anxiety*, *GSI* and *GSI-18*. Older participants reached lower sum scores than participants aged 18–25 years in *Interpersonal Sensitivity*, *Depression*, *Anxiety*, *Hostility*, *Psychoticism* and *GSI*. Regarding the language region, participants from the French speaking areas reached lower sum scores than those from the German speaking area on *Depression*, but higher scores on *Anxiety*. Participants from the Italian speaking area reached higher scores than those from the German speaking area on *Somatization (*also *Somatization 6-items)*, *Anxiety*, *Hostility* and *GSI-18*. Participants from the French speaking area reached lower scores than those from the German speaking area on *Depression and Anxiety*. Participants with a higher education reached lower sum scores than those with compulsory education on *Somatization (*also *Somatization 6-items)*, *Obsessive-Compulsive Tendencies*, *Depression*, *Phobic Anxiety*, *Paranoid Ideation*, *Psychoticism*, *GSI* and *GSI-18*. Employed participants reached lower sum scores than unemployed on *Obsessive-Compulsive Tendencies*, *Interpersonal Sensitivity*, *Depression*, *Phobic Anxiety*, *Psychoticism*, *GSI* and *GSI-18*. Finally, there was no difference between participants with and without migration background, if the other characteristics were accounted for (S12 Table in (S4 Appendix)).

**Table 6:** Associations of socio-demographic characteristics with the BSI Global Severity Index (Sum score).

	Univariable linear regression				Multivariable linear regression*			
	Coef	95% CI	p	Coef	95% CI	p
**Sex**	F(1,1231) = 10.84; p<**0.001**							
Male (baseline)	16.6	14.9	18.3					
Female	3.8	1.5	6.1	0.001	3.0	0.7	5.3	**0.010**
**Age group**	F(5,1227) = 2.560; p = **0.026**							
18–25 years (baseline)	26.3	21.4	31.3					
26–35 years	-7.3	-13.0	-1.6	0.012	-2.8	-9.4	3.8	0.407
36–45 years	-8.4	-13.9	-2.9	0.003	-3.0	-9.2	3.3	0.350
46–55 years	-9.0	-14.5	-3.5	0.001	-4.0	-10.5	2.5	0.229
56–65 years	-8.5	-14.0	-2.9	0.003	-5.8	-11.8	0.2	0.058
66–75 years	-9.5	-15.0	-4.1	0.001	-8.3	-15.2	-1.5	**0.016**
**Language Region**	F(2,1230) = 3.15; p = 0.043							
German(baseline)	18.2	17.0	19.5					
French	-0.6	-3.4	2.2	0.67	-0.7	-3.6	2.1	0.618
Italian	8.7	1.7	15.8	0.015	5.4	-1.1	11.9	0.106
**Education**	F(3,1157) = 6.960; p<**0.001**							
Compulsory schooling (baseline)	25.6	20.4	30.7					
Vocational training	-6.0	-11.4	-0.6	0.03	-4.0	-9.4	1.4	0.145
Upper secondary education	-9.9	-15.4	-4.5	0	-6.7	-12.2	-1.2	**0.017**
University education	-9.7	-15.2	-4.1	0.001	-6.4	-12.0	-0.7	**0.026**
**Employment**	F(2,1199) = 7.910; p<**0.001**							
Unemployed (baseline)	26.6	22.0	31.1					
Employed	-9.3	-14.1	-4.6	0	-6.9	-12.6	-1.2	**0.018**
Retired	-9.7	-14.7	-4.8	0	-4.1	-10.6	2.4	0.216
**Migration background**	F(1,1231) = 0.920; p = 0.339							
No migration background (baseline)	18.2	16.9	19.4					
Migration background	1.3	-1.4	4.0	0.339				

*Test statistic for the multivariable linear regression model: F(11, 1144) = 4.880; p < 0.001; p-values <0.05 in bold.

Abbreviations: BSI Brief symptom Inventory, Coef coefficient, CI confidence interval

**Fig 1:**
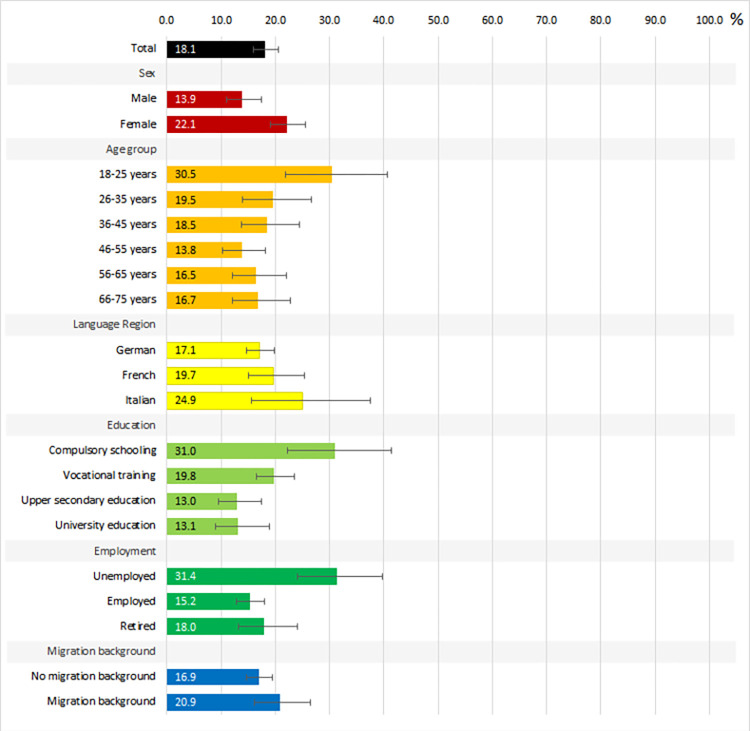
Proportion of cases with significant distress (with 95% confidence intervals).

***Cases with psychological distress***: A total of 18.1% participants (CI: 16.0–20.5) were considered *cases with psychological distress* (Fig 1, S13 Table in (S4 Appendix)). Females (22.1%, CI:19.1–25.5, p<0.001), young people (18–25 years, 30.5%, CI:21.9–40.7, p = 0.019), those with compulsory schooling only (31.0%, CI:22.2–41.4, p<0.001), and unemployed participants (31.4%, CI:24.0–39.8, p<0.001) were more likely to be *cases with psychological distress*.

### Aim 4: Comparison of the Swiss and the German norm populations

The means of the Swiss general population differ little when compared to the three German norm populations. The least differences seem to be with the oldest norms presented by Franke, 2000 [20] (S14 Table in S5 Appendix). The T-scores, when different standardizations are applied to the Swiss general population data, are statistically different (S15 Table in S5 Appendix). However, the differences are very small and not of clinical relevance, and again the least differences can be found between the Swiss T-standardization and the one presented by Franke, 2000 [20].

## Discussion

The BSI is a commonly used patient reported outcome measure (PROM) in research and treatment of inpatients and outpatients in Switzerland. Our study presents normative data on psychological distress assessed by the BSI based on a representative sample of the Swiss general population. We have developed a T-standardization which can be used in other Swiss samples. Finally, we provide descriptive comparison data on the Swiss general population and a clinical sample of psychotherapy outpatients to be used for other Swiss and international studies.

The BSI showed good psychometric properties with a medium to high reliability on all scales. Reliability was slightly higher than in the German norm population [20] and slightly lower than in Derogatis’ original sample of psychiatry outpatients [18]. However, CFA showed only mediocre model fit similar to previous studies [43, 45, 46]. Although not tested in our study, test-retest reliability had been high in previous general population studies [20], and fair in a sample of college and high school students for short periods of time (7 days) [21]. Similar to previous studies, we found that BSI scales and the *GSI* had a left-skewed distribution [20, 47], indicating that the majority of participants in the general population do not report symptoms of distress or only few.

Our sample of psychotherapy patients had considerably higher scores on all scales compared to the general population sample, with many patients reaching a T≥63 indicating scale-caseness. A total of 75% were considered cases with psychological distress (reaching T≥63 on the *GSI*, or T≥63 on at least two of the scales). This further supports the good validity of the BSI and its usefulness in the clinical setting and for research [48].

We also present normative data for various socio-demographic subgroups. Overall, females reported higher distress than males, a result that has been found in many other studies using the BSI (e.g. in general populations [20, 49, 50], high school and college athletes [21], cancer patients [36] and cancer survivors [10]). A recent Canadian study indicated that low decision authority, lower self-esteem (which is more prevalent among women), and work-family-conflicts might be some of the reasons why women report higher levels of psychological distress [51]. Similarly, younger participants, those with compulsory schooling only and unemployed participate reported higher distress and were more likely to reach potentially clinical levels of distress (caseness) [2, 22, 52, 53]. While the effect of age might be explained by cognitive and emotional development but also differences in developmental tasks which are predominant in adolescence and young adulthood [54], others have found that a cohort effect related to political or global events might be important [55]. Regarding socio-economic characteristics, there is evidence based on a systematic review and meta-analyses that they influence psychological distress on various levels, such as experienced stress on the individual level or social comparisons on the neighborhood level [56]. The detailed information provided in S8 Table in (S3 Appendix) will help future studies to compare their results with various subgroups of the Swiss general population.

In previous studies conducted in Switzerland, the German norm-sample from Franke, 2000 [20] has frequently been used as a comparison in lack of Swiss normative data. Our study showed that there are small differences between the scores of the Swiss general population and German norm populations [20, 29, 30]. Compared to the normative data from Franke, 2000 [20], the Swiss general population tends to reach slightly higher scores for certain scales (*Paranoid Ideation*, *Psychoticism* and especially the *GSI*), indicating a slightly higher level of distress in the Swiss general population. Compared to the two other German norm samples [29, 30], the Swiss scores tend to be slightly lower especially regarding *Somatization* and *Obsessive-Compulsive Tendencies*. The American Psychological Association highlights the importance of using norms related to age, gender, ethnicity, primary language etc. [57] The use of Swiss normative data is thus essential to allow meaningful comparisons in the Swiss research and clinical setting, especially considering the different language regions in Switzerland.

Screening for psychological distress using the BSI is standard practice in Swiss psychiatric clinics to measure self-reported symptom severity at entry and exit of inpatient treatment (www.anq.ch/en/departments/psychiatry). This helps to evaluate the change of symptom burden and the effectiveness of treatment. However, screening was also shown to identify a considerable number of previously unidentified and/or untreated patients in primary care with about 1 in 10 of them reporting severe distress or suicidal ideation [58]. Around 18% of our sample were considered cases with psychological distress. While the caseness criterion must not be interpreted as clear indicator of an individual having a psychiatric diagnosis or being in need of treatment, it provides a probabilistic value to maximize case identification [59]. The number of people in the Swiss general population who might profit from further evaluation and potentially psychological or psychiatric support or treatment is rather high, especially in the younger, less educated, and unemployed population. Offering screening and easy access to psychological support in primary care, schools, or regional employment centers might help to support individuals in need for psychological support at an early stage.

### Methodological considerations

The relatively low response rate to our questionnaire of 22.2% is a limitation of our study. People with a very high level of distress might not have participated due to their problems, which would result in an underestimation of the level of psychological distress in the Swiss general population. On the other hand, people experiencing distress might have been more motivated to share their experience making it more likely that they participate. Further, participation was lower in young people, males and those with another than Swiss nationality. This is similar to other health-related surveys [60, 61]. However, one of these studies found that the variation in the measured outcome remained fairly stable despite different response rates [60]. The questionnaire was part of a larger study, which included various questionnaires on health and well-being of the Swiss general population [62–64]. The rather long questionnaire might have affected people’s willingness to participate. The reliability of two scales was below 0.7 (*Hostility* α = 0.698; *Psychoticism* α = 0.679), and might just be considered acceptable. However, this is similar to various other studies [29]. Finally, we were not able to assess criterion validity in detail. However, scores of psychotherapy patients were clearly higher than in the general population and a larger proportion of these patients were considered cases with psychological distress on the BSI. This finding supports the validity of the BSI in measuring psychological distress.

A strength of our study is the large, representative, and population-based sample we have received from the SFSO. Despite a relatively low response rate we were able to use statistical weighting with the information provided by the SFSO and work with a representative sample of the Swiss general population. The data was collected between May 2015 and June 2016, but there is generally no indication for changes in the prevalence of psychological distress over time [65]. The COVID-19 pandemic had an obvious impact on mental health in the general population [66], but first studies indicate decrease of mental health problems to pre-COVID-19 levels [67]. We therefore expect that our findings can be considered valid for Switzerland for now and the future.

## Conclusion

This is the first study that presents detailed normative data on psychological distress as assessed by the Brief Symptom Inventory for the Swiss general population including three different language regions (German, French, and Italian). This information will be helpful for clinical applications and research in the Swiss context and can be used for international comparisons.

## Supporting information

S1 Checklist STROBE statement—Checklist of items that should be included in reports of observational studies. (DOCX)

S2 Checklist (PDF)

S1 Appendix (PDF)

S2 Appendix (PDF)

S3 Appendix (PDF)

S4 Appendix (PDF)

S5 Appendix (PDF)
