# Supplementary material for: The Brief Symptom Inventory in the Swiss general population: Presentation of norm scores and predictors of psychological distress
Source: PLoS One. 2024 Jul 3;19(7):e0305192. doi: 10.1371/journal.pone.0305192 (PMC11221686; doi:10.1371/journal.pone.0305192)
Supplement: S2 Checklist — (PDF) [file pone.0305192.s002.pdf]

S 16 Table: STROBE Statement—checklist of items that should be included in reports of observational studies

	Item No.	Recommendation	Page No.	Relevant text from manuscript
**Title and abstract**	1	(*a*) Indicate the study's design with a commonly used term in the title or the abstract		
		(*b*) Provide in the abstract an informative and balanced summary of what was done and what was found	3	Using a cross-sectional study design,
**Introduction**				
Background/rationale	2	Explain the scientific background and rationale for the investigation being reported	4-6	
Objectives	3	State specific objectives, including any prespecified hypotheses	6-7	Our objective was to provide normative data for the BSI and the BSI-18 for the Swiss general population. Using a representative sample of the Swiss general population, we aimed to: 1) present psychometric properties of the BSI for Switzerland, 2) develop Swiss T-standardization for the BSI (nine scales and three global indices) and validate it using a clinical sample of psychotherapy outpatients, and 3) describe psychological distress in the Swiss general population, subgroups with different characteristics, and the clinical sample. In addition to the original BSI, we also present these results for the BSI-18. 4) Finally, we compared the means and T-standardized scores (according to the German standardizations) of the Swiss general population to different German norm populations.
**Methods**				
Study design	4	Present key elements of study design early in the paper	8	The representative sample for this cross-sectional study was provided by the Swiss Federal Statistical Office (SFSO)…
Setting	5	Describe the setting, locations, and relevant dates, including periods of recruitment, exposure, follow-up, and data collection	8-9	
Participants	6	(*a*) *Cohort study*—Give the eligibility criteria, and the sources and methods of selection of participants. Describe methods of follow-up<br>*Case-control study*—Give the eligibility criteria, and the sources and methods of case ascertainment and control selection. Give the rationale for the choice of cases and controls<br>*Cross-sectional study*—Give the eligibility criteria, and the sources and methods of selection of participants	8	

		(*b*) *Cohort study*—For matched studies, give matching criteria and number of exposed and unexposed<br>*Case-control study*—For matched studies, give matching criteria and the number of controls per case	
Variables	7	Clearly define all outcomes, exposures, predictors, potential confounders, and effect modifiers. Give diagnostic criteria, if applicable	9-10
Data sources/ measurement	8*	For each variable of interest, give sources of data and details of methods of assessment (measurement). Describe comparability of assessment methods if there is more than one group	*9-10*
Bias	9	Describe any efforts to address potential sources of bias	8-10
Study size	10	Explain how the study size was arrived at	13

Quantitative variables	11	Explain how quantitative variables were handled in the analyses. If applicable, describe which groupings were chosen and why	7-10
Statistical methods	12	(*a*) Describe all statistical methods, including those used to control for confounding	9-12
		(*b*) Describe any methods used to examine subgroups and interactions	10-12
		(*c*) Explain how missing data were addressed	9-10
		(*d*) *Cohort study*—If applicable, explain how loss to follow-up was addressed<br>*Case-control study*—If applicable, explain how matching of cases and controls was addressed<br>*Cross-sectional study*—If applicable, describe analytical methods taking account of sampling strategy	na
		(*e*) Describe any sensitivity analyses	na
**Results**			
Participants	13*	(a) Report numbers of individuals at each stage of study—eg numbers potentially eligible, examined for eligibility, confirmed eligible, included in the study, completing follow-up, and analysed	13
		(b) Give reasons for non-participation at each stage	na
		(c) Consider use of a flow diagram	na
Descriptive data	14*	(a) Give characteristics of study participants (eg demographic, clinical, social) and information on exposures and potential confounders	14, Table 1
		(b) Indicate number of participants with missing data for each variable of interest	Table 2
		(c) *Cohort study*—Summarise follow-up time (eg, average and total amount)	

Outcome data	15*	*Cohort study*—Report numbers of outcome events or summary measures over time	
		Case-control study—Report numbers in each exposure category, or summary measures of exposure	
		Cross-sectional study—Report numbers of outcome events or summary measures	*Table 2, 4, 5 (appendices)*
Main results	16	(*a*) Give unadjusted estimates and, if applicable, confounder-adjusted estimates and their precision (eg, 95% confidence interval). Make clear which confounders were adjusted for and why they were included	All of results
		(*b*) Report category boundaries when continuous variables were categorized	10
		(*c*) If relevant, consider translating estimates of relative risk into absolute risk for a meaningful time period	na

Continued on next page

Other analyses	17	Report other analyses done—eg analyses of subgroups and interactions, and sensitivity analyses	Extensive appendices
**Discussion**			
Key results	18	Summarise key results with reference to study objectives	23
Limitations	19	Discuss limitations of the study, taking into account sources of potential bias or imprecision. Discuss both direction and magnitude of any potential bias	26-27
Interpretation	20	Give a cautious overall interpretation of results considering objectives, limitations, multiplicity of analyses, results from similar studies, and other relevant evidence	23-26
Generalisability	21	Discuss the generalisability (external validity) of the study results	26
**Other information**			
Funding	22	Give the source of funding and the role of the funders for the present study and, if applicable, for the original study on which the present article is based	Reported in submission form as requested by PLOS One

*Give information separately for cases and controls in case-control studies and, if applicable, for exposed and unexposed groups in cohort and cross-sectional studies.

Note: An Explanation and Elaboration article discusses each checklist item and gives methodological background and published examples of transparent reporting. The STROBE checklist is best used in conjunction with this article (freely available on the Web sites of PLoS Medicine at http://www.plosmedicine.org/, Annals of Internal Medicine at http://www.annals.org/, and Epidemiology at http://www.epidem.com/). Information on the STROBE Initiative is available at www.strobe-statement.org.
