# Supplementary material for: The Brief Symptom Inventory in the Swiss general population: Presentation of norm scores and predictors of psychological distress
Source: PLoS One. 2024 Jul 3;19(7):e0305192. doi: 10.1371/journal.pone.0305192 (PMC11221686; doi:10.1371/journal.pone.0305192)
Supplement: S1 Appendix — (PDF) [file pone.0305192.s003.pdf]

Psychometric properties of the Brief Symptom Inventory in the Swiss general population: Presentation of norm scores and predictors of psychological distress

Gisela Michel [1*], Julia Baenziger [1], Jeannette Brodbeck [2], Luzius Mader [1,3,4], Claudia Kuehni [3,5], Katharina Roser [1]

[1] Faculty of Health Sciences and Medicine, University of Lucerne, Alpenquai 4, 6005 Lucerne, Switzerland; E-mail: gisela.michel@unilu.ch, julia.baenziger@outlook.com, katharina.roser@unilu.ch
[2] Institute of Psychology, University of Bern, Fabrikstrasse 8, 3012 Bern, Switzerland. E-mail: jeannette.brodbeck@unibe.ch
[3] Institute for Social and Preventive Medicine, University of Bern, Mittelstrasse 43, 3012 Bern, Switzerland. E-mail: claudia.kuehni@ispm.unibe.ch
[4] Cancer Registry Bern-Solothurn, University of Bern, Murtenstrasse 31, 3008 Bern, Switzerland. E-mail: luzius.mader@unibe.ch
[5] Pediatric Hematology and Oncology, University Children's Hospital Bern, University of Bern, Freiburgstrasse 15, 3010 Bern, Bern, Switzerland.

*Corresponding author: Gisela Michel, Faculty of Health Sciences and Medicine, University of Lucerne, Alpenquai 4, 6005 Lucerne, Switzerland, E-mail: gisela.michel@unilu.ch

Appendix A: Supplemental Tables

Content:

S1 Table: Comparison between responders and non-responders of the Swiss general population on characteristics provided by the Swiss Federal Statistical Office 2

S2 Table: Description of the clinical sample 3

S3 Table: Psychometric properties of the Brief Symptom Inventory (BSI) in the Swiss general population 4

Confirmatory factor analysis of the Brief Symptom Inventory 8

S4 Table: Goodness-of-fit indices for the confirmatory factor analysis of the original 9 factor solution of the of the BSI 8

S5 Table: Unstandardized and standardized coefficients of the CFA 9

S6 Table: External validation of the Swiss T-standardization with a clinical sample of psychotherapy patients (N=1005): Comparison of Caseness in outpatients with different diagnoses for the different BSI scales, the GSI, and overall caseness 11

S1 Table: Comparison between responders and non-responders of the Swiss general population on characteristics provided by the Swiss Federal Statistical Office

		Response status				
	Total	No		Yes		
		N	%	N	%	p-value
**Total**	5644	4389	77.8	1255	22.2	
**Sex**						<0.001
Male	2822	2298	52.4	524	41.8	
Female	2822	2091	47.6	731	58.2	
**Age (years)**						<0.001
18-25	551	458	10.4	93	7.4	
26-35	1053	885	20.2	168	13.4	
36-45	1039	804	18.3	235	18.7	
46-55	1234	947	21.6	287	22.9	
56-65	967	726	16.5	241	19.2	
66-75	800	569	13.0	231	18.4	
**Language of questionnaire**						0.162
German	4096	3177	72.4	919	73.2	
French	1286	1018	23.2	268	21.4	
Italian	262	194	4.4	68	5.4	
**Nationality**						<0.001
Other	1385	1222	27.8	163	13.0	
Swiss	4257	3165	72.1	1092	87.0	
Missing	2	2	0.0	0	0.0	
**Marital status**						0.003
Single	2010	1604	36.5	406	32.4	
Married	2972	2298	52.4	674	53.7	
Divorced or widowed	662	487	11.1	175	13.9	

p-values from Chi^2 tests

S2 Table: Description of the clinical sample

	N	%
**Total**	**3152**	
**Information on sex available**	761	
Male	331	43.5
Female	430	56.5
**Diagnosis**		
**Total patients with a diagnosis**	1005	100
Depression	506	50.3
Social phobia	211	21.0
Agoraphobia and panic disorder	129	12.8
Generalized Anxiety Disorder	21	2.1
Phobia	73	7.3
Eating disorder	69	6.9
Somatization	65	6.5
Substance abuse	102	10.1
Compulsory disorder	53	5.3
Post-traumatic stress disorder	38	3.8
Sexual disorder	27	2.7
Personality disorder	61	6.1
Sleep disorder	16	1.6
Psychotic disorder	7	0.7
Adjustment disorder	149	14.8
**Number of diagnosis**		
1	590	58.7
2	319	31.7
3	87	8.7
4	7	0.7
5	2	0.2

S3 Table: Psychometric properties of the Brief Symptom Inventory (BSI) in the Swiss general population

Item	N	Item-scale correlation	Item-rest correlation	Average inter-item correlation	Alpha<br>For each item: alpha without the respective item
**BSI (53 items)**					
**Somatization**				**0.258**	**0.71**
Item 2	1234	0.577	0.411	0.26	0.68
Item 7	1236	0.506	0.331	0.28	0.69
Item 23	1237	0.579	0.382	0.26	0.68
Item 29	1237	0.576	0.421	0.26	0.68
Item 30	1235	0.643	0.439	0.25	0.67
Item 33	1237	0.646	0.448	0.25	0.67
Item 37	1235	0.683	0.496	0.24	0.65
**Obsessive-compulsive tendencies**				**0.233**	**0.80**
Item 5	1229	0.705	0.541	0.41	0.78
Item 15	1237	0.716	0.546	0.41	0.78
Item 26	1234	0.648	0.482	0.44	0.79
Item 27	1233	0.719	0.565	0.41	0.77
Item 32	1230	0.672	0.553	0.41	0.78
Item 36	1237	0.810	0.694	0.37	0.74
**Interpersonal sensitivity**				**0.275**	**0.80**
Item 20	1236	0.803	0.586	0.53	0.77
Item 21	1238	0.797	0.630	0.50	0.74
Item 22	1236	0.836	0.675	0.47	0.71
Item 42	1238	0.732	0.580	0.53	0.77
**Depression**				**0.436**	**0.82**
Item 9	1235	0.544	0.450	0.48	0.82
Item 16	1237	0.796	0.639	0.42	0.78
Item 17	1232	0.840	0.731	0.39	0.75
Item 18	1237	0.694	0.549	0.45	0.79
Item 35	1236	0.725	0.571	0.44	0.79
Item 50	1237	0.739	0.616	0.42	0.78
**Anxiety**				**0.397**	**0.77**
Item 1	1236	0.781	0.579	0.40	0.74
Item 12	1235	0.640	0.516	0.40	0.75
Item 19	1236	0.723	0.596	0.37	0.73
Item 38	1233	0.791	0.617	0.39	0.72
Item 45	1236	0.634	0.529	0.39	0.75
Item 49	1238	0.610	0.449	0.43	0.76
**Hostility**				**0.338**	**0.70**
Item 6	1236	0.798	0.531	0.33	0.63
Item 13	1235	0.788	0.587	0.31	0.58
Item 40	1237	0.533	0.371	0.36	0.68
Item 41	1236	0.536	0.396	0.35	0.68
Item 46	1232	0.702	0.504	0.33	0.63

S3 Table contd.

Item	N	Item-scale correlation	Item-rest correlation	Average inter-item covariance	**Alpha** For each item: alpha without the respective item
**Phobic anxiety**				**0.381**	**0.71**
Item 8	1238	0.707	0.548	0.36	0.64
Item 28	1235	0.688	0.562	0.35	0.66
Item 31	1238	0.695	0.519	0.37	0.65
Item 43	1238	0.779	0.492	0.40	0.70
Item 47	1236	0.649	0.430	0.43	0.69
**Paranoid ideation**				**0.396**	**0.76**
Item 4	1237	0.649	0.484	0.42	0.74
Item 10	1236	0.702	0.483	0.42	0.74
Item 24	1237	0.698	0.527	0.40	0.72
Item 48	1234	0.757	0.581	0.38	0.70
Item 51	1238	0.780	0.601	0.37	0.69
**Psychoticism**				**0.303**	**0.68**
Item 3	1232	0.527	0.340	0.34	0.67
Item 14	1237	0.783	0.513	0.28	0.60
Item 34	1232	0.516	0.350	0.34	0.67
Item 44	1234	0.747	0.526	0.28	0.58
Item 53	1236	0.710	0.503	0.28	0.60

S3 Table contd.

Item	N	Item-scale correlation	Item-rest correlation	Average inter-item correlation	Alpha<br>For each item: alpha without the respective item
**GSI**				**0.284**	**0.95**
Item 1	1236	0.586	0.553	0.28	0.95
Item 2	1234	0.412	0.388	0.29	0.95
Item 3	1232	0.401	0.382	0.29	0.95
Item 4	1237	0.478	0.452	0.29	0.95
Item 5	1229	0.533	0.502	0.28	0.95
Item 6	1236	0.590	0.558	0.28	0.95
Item 7	1236	0.321	0.296	0.29	0.95
Item 8	1238	0.477	0.459	0.29	0.95
Item 9	1235	0.382	0.366	0.29	0.95
Item 10	1236	0.497	0.463	0.29	0.95
Item 11	1233	0.484	0.462	0.29	0.95
Item 12	1235	0.559	0.542	0.28	0.95
Item 13	1235	0.605	0.581	0.28	0.95
Item 14	1237	0.683	0.659	0.28	0.95
Item 15	1237	0.666	0.641	0.28	0.95
Item 16	1237	0.673	0.647	0.28	0.95
Item 17	1232	0.702	0.681	0.28	0.95
Item 18	1237	0.573	0.550	0.28	0.95
Item 19	1236	0.663	0.645	0.28	0.95
Item 20	1236	0.689	0.664	0.28	0.95
Item 21	1238	0.647	0.625	0.28	0.95
Item 22	1236	0.702	0.680	0.28	0.95
Item 23	1237	0.439	0.412	0.29	0.95
Item 24	1237	0.594	0.571	0.28	0.95
Item 25	1238	0.471	0.432	0.29	0.95
Item 26	1234	0.533	0.505	0.28	0.95
Item 27	1233	0.584	0.556	0.28	0.95
Item 28	1235	0.401	0.386	0.29	0.95
Item 29	1237	0.340	0.316	0.29	0.95
Item 30	1235	0.390	0.359	0.29	0.95
Item 31	1238	0.491	0.473	0.29	0.95
Item 32	1230	0.562	0.541	0.28	0.95
Item 33	1237	0.403	0.372	0.29	0.95
Item 34	1232	0.422	0.406	0.29	0.95
Item 35	1236	0.621	0.597	0.28	0.95
Item 36	1237	0.665	0.641	0.28	0.95
Item 37	1235	0.547	0.521	0.28	0.95

S3 Table contd.

Item	N	Item-scale correlation	Item-rest correlation	Average inter-item correlation	**Alpha** For each item: alpha without the respective item
Item 38	1233	0.656	0.630	0.28	0.95
Item 39	1234	0.420	0.390	0.29	0.95
Item 40	1237	0.430	0.412	0.29	0.95
Item 41	1236	0.415	0.399	0.29	0.95
Item 42	1238	0.662	0.645	0.28	0.95
Item 43	1238	0.534	0.505	0.28	0.95
Item 44	1234	0.600	0.577	0.28	0.95
Item 45	1236	0.581	0.566	0.28	0.95
Item 46	1232	0.543	0.520	0.28	0.95
Item 47	1236	0.591	0.573	0.28	0.95
Item 48	1234	0.572	0.544	0.28	0.95
Item 49	1238	0.543	0.522	0.28	0.95
Item 50	1237	0.673	0.655	0.28	0.95
Item 51	1238	0.602	0.574	0.28	0.95
Item 52	1236	0.621	0.601	0.28	0.95
Item 53	1236	0.630	0.611	0.28	0.95
**BSI-18**					
**Somatization (6 items)**				**0.254**	**0.67**
Item 2	1234	0.602	0.407	0.25	0.63
Item 7	1236	0.537	0.333	0.27	0.65
Item 23	1237	0.594	0.360	0.26	0.64
Item 29	1237	0.582	0.396	0.25	0.63
Item 33	1237	0.662	0.425	0.25	0.62
Item 37	1235	0.703	0.483	0.23	0.59
**GSI-18**				**0.287**	**0.88**
Item 2	1234	0.488	0.422	0.29	0.87
Item 7	1236	0.394	0.325	0.30	0.88
Item 23	1237	0.484	0.406	0.29	0.87
Item 29	1237	0.393	0.325	0.30	0.88
Item 33	1237	0.460	0.373	0.30	0.88
Item 37	1235	0.594	0.520	0.29	0.87
Item 9	1235	0.421	0.376	0.30	0.87
Item 16	1237	0.689	0.611	0.28	0.87
Item 17	1232	0.740	0.680	0.28	0.86
Item 18	1237	0.585	0.514	0.29	0.87
Item 35	1236	0.625	0.553	0.28	0.87
Item 50	1237	0.635	0.574	0.28	0.87
Item 1	1236	0.644	0.551	0.28	0.87
Item 12	1235	0.594	0.543	0.28	0.87
Item 19	1236	0.689	0.640	0.28	0.87
Item 38	1233	0.686	0.609	0.28	0.87
Item 45	1236	0.589	0.545	0.28	0.87
Item 49	1238	0.544	0.478	0.29	0.87

Abbreviations: BSI Brief Symptom Inventory, BIS-18 Brief Symptom Inventory 18 items, GSI Global Severity Index, GSI-18 Global Severity Index for the Brief Symptom Inventory 18 (only including items of the Somatization (6 items), Depression and Anxiety scale

Confirmatory factor analysis of the Brief Symptom Inventory

We performed a confirmatory factor analysis (CFA) using the SEM command and maximum likelihood estimation in STATA 18.0. We present the following indices to evaluate model fit: the Comparative Fit Index (CFI), the Tucker Lewis Index (TLI), and the Root Mean Square Error of Approximation (RMSEA). For the Comparative Fit Index (CFI), a value of >0.97 indicates good and 0.95-0.97 adequate model fit[1]; a Tucker-Lewis Index (TLI)-value of 0.95 and above indicates good model fit[2]; Root Mean Square Error of Approximation (RMSEA)-values under 0.01 indicates excellent, 0.05 good, and 0.08 mediocre fit [3]; Standardized Root Mean Square Residual (SRMR)-value less than <0.05 indicates good and 0.05-0.10 adequate model fit [1].

Our goodness of fit indices (S4 Table) show that the nine scales only have a poor to adequate/mediocre fit for our data. This is in accordance with other studies (e.g. [4-6]) which could not replicate the original factorial structure satisfactorily.

S4 Table: Goodness-of-fit indices for the confirmatory factor analysis of the original 9 factor solution of the of the BSI

Chi Square (df); p-value	6091 (1091); <0.001	
CFI	0.782	Poor fit
TLI	0.765	Poor fit
RMSEA (90% CI)	0.063 (0.61-0.65)	Mediocre fit
SRMR	0.059	Adequate fit

CFI Comparative Fit Index; TLI Tucker Lewis Index; RMSEA Root Mean Square Error of Approximation; SRMR Standardized Root Mean Square Residual

S5 Table: Unstandardized and standardized coefficients of the CFA

Scale / Item	Unstandardized Coefficient	95% CI		Standardized Coefficient	95% CI	
**Somatization**						
Item 2	1.00	(constrained)		0.52	0.46	0.57
Item 7	0.66	0.53	0.79	0.37	0.31	0.42
Item 23	1.14	0.97	1.32	0.52	0.47	0.57
Item 29	0.89	0.74	1.03	0.50	0.44	0.55
Item 30	1.27	1.07	1.48	0.52	0.46	0.57
Item 33	1.16	0.97	1.34	0.52	0.46	0.57
Item 37	1.43	1.22	1.63	0.62	0.57	0.66
**Obsessive-compulsive tendencies**						
Item 5	1.00	(constrained)		0.58	0.54	0.63
Item 15	1.21	1.07	1.35	0.68	0.64	0.71
Item 26	0.83	0.72	0.94	0.52	0.47	0.57
Item 27	1.10	0.97	1.23	0.65	0.61	0.69
Item 32	0.73	0.64	0.82	0.61	0.57	0.65
Item 36	1.28	1.15	1.40	0.75	0.72	0.78
**Interpersonal sensitivity**						
Item 20	1.00	(constrained)		0.69	0.66	0.73
Item 21	0.84	0.77	0.92	0.71	0.68	0.75
Item 22	0.96	0.88	1.05	0.76	0.73	0.79
Item 42	0.59	0.53	0.64	0.64	0.6	0.67
**Depression**						
Item 9	1.00	(constrained)		0.41	0.36	0.46
Item 16	4.41	3.77	5.06	0.76	0.73	0.79
Item 17	3.90	3.34	4.47	0.77	0.74	0.8
Item 18	2.50	2.10	2.91	0.59	0.55	0.63
Item 35	2.99	2.53	3.46	0.61	0.57	0.65
Item 50	2.95	2.51	3.39	0.71	0.67	0.74
**Anxiety**						
Item 1	1.00	(constrained)		0.58	0.53	0.62
Item 12	0.48	0.42	0.55	0.56	0.52	0.6
Item 19	0.68	0.60	0.75	0.67	0.63	0.71
Item 38	1.03	0.92	1.15	0.64	0.60	0.68
Item 45	0.46	0.40	0.52	0.62	0.58	0.66
Item 49	0.51	0.44	0.58	0.52	0.47	0.56
**Hostility**						
Item 6	1.00	(constrained)		0.69	0.65	0.73
Item 13	0.79	0.71	0.87	0.69	0.65	0.73
Item 40	0.26	0.21	0.30	0.39	0.34	0.45
Item 41	0.19	0.16	0.23	0.36	0.31	0.42
Item 46	0.55	0.48	0.62	0.58	0.53	0.62
**Phobic anxiety**						
Item 8	1.00	(constrained)		0.68	0.64	0.72
Item 28	0.86	0.77	0.94	0.68	0.64	0.72

Item 31	1.07	0.95	1.19	0.65	0.61	0.7
Item 43	1.57	1.38	1.76	0.57	0.52	0.62
Item 47	0.90	0.77	1.03	0.52	0.47	0.57
**Paranoid ideation**						
Item 4	1.00	(constrained)		0.55	0.5	0.59
Item 10	1.40	1.20	1.59	0.54	0.5	0.59
Item 24	1.39	1.22	1.56	0.68	0.64	0.72
Item 48	1.61	1.42	1.80	0.67	0.63	0.71
Item 51	1.71	1.51	1.92	0.68	0.64	0.72
**Psychoticism**						
Item 3	1.00	(constrained)		0.35	0.29	0.4
Item 14	3.97	3.29	4.65	0.72	0.69	0.75
Item 34	1.00	0.79	1.22	0.38	0.33	0.43
Item 44	2.74	2.25	3.23	0.59	0.55	0.63
Item 53	2.17	1.77	2.56	0.56	0.52	0.61

References

1. Schermelleh-Engel, K., H. Moosbrugger, and H. Müller, *Evaluating the Fit of Structural Equation Models: Tests of Significance and Descriptive Goodness-of-Fit Measures.* Methods of Psychological Research, 2003. **8**(2): p. 23-74.
2. Hu, L.T. and P.M. Bentler, *Evaluating model fit*, in *Structural equation modeling: Concepts, issues and application*, R.H. Hoyle, Editor. 1995, Sage: Thousand Oaks, CA. p. 77-99.
3. MacCallum, R.C., M.W. Browne, and H.M. Sugawara, *Power analysis and determination of sample size for covariance structure modeling.* Psychological Methods, 1996. **1**(2): p. 130-149.
4. Schwannauer, M. and P. Chetwynd, *The Brief Symptom Inventory: A validity study in two independent Scottish samples.* Clinical Psychology & Psychotherapy, 2007. **14**(3): p. 221-228.
5. Brodbeck, J., et al., *The structure of psychopathological symptoms and the associations with DSM-diagnoses in treatment seeking individuals.* Compr Psychiatry, 2014. **55**(3): p. 714-26.
6. Geisheim, C., et al., *Das Brief Symptom Inventory (BSI) als Instrument zur Qualitätssicherung in der Psychotherapie.* Diagnostica, 2002. **48**(1): p. 28-36.

S6 Table: External validation of the Swiss T-standardization with a clinical sample of psychotherapy patients (N=1005): Comparison of Caseness in outpatients with different diagnoses for the different BSI scales, the GSI, and overall caseness

Diagnosis	Total	Depression			Social phobia			Agoraphbia / Panic disorder			Generalized Anxiety Disorder			Phobia			Somatization			Compulsory disorder			Psychosis		
BSI-scale	n	n	%	p	n	%	p	n	%	p	n	%	p	n	%	p	n	%	p	n	%	p	n	%	p
Total		506			211			129			21			73			65			53			7		
**Somatization**				**0.005**			**0.037**			**<0.001**			0.685			0.731			**0.002**			0.262			0.120
T<63	570	265	52.4		133	63.0		38	29.5		11	52.4		40	54.8		25	38.5		34	64.2		6	85.7	
T≥63	435	241	47.6		78	37.0		91	70.5		10	47.6		33	45.2		40	61.5		19	35.8		1	14.3	
**Obsessive-compulsive tendencies**				**<0.001**			0.898			0.472			0.803			**<0.001**			0.329			0.235			0.240
T<63	361	116	22.9		75	35.5		50	38.8		7	33.3		43	58.9		27	41.5		15	28.3		4	57.1	
T≥63	644	390	77.1		136	64.5		79	61.2		14	66.7		30	41.1		38	58.5		38	71.7		3	42.9	
**Interpersonal sensitivity**				**<0.001**			**<0.001**			**0.022**			0.649			**<0.001**			0.394			0.163			0.069
T<63	383	143	28.3		54	25.6		61	47.3		7	33.3		46	63.0		28	43.1		25	47.2		5	71.4	
T≥63	622	363	71.7		157	74.4		68	52.7		14	66.7		27	37.0		37	56.9		28	52.8		2	28.6	
**Depression**				**<0.001**			0.358			0.142			0.961			**0.002**			0.590			0.565			0.191
T<63	340	90	17.8		77	36.5		51	39.5		7	33.3		37	50.7		20	30.8		16	30.2		4	57.1	
T≥63	665	416	82.2		134	63.5		78	60.5		14	66.7		36	49.3		45	69.2		37	69.8		3	42.9	
**Anxiety**				**<0.001**			0.431			**<0.001**			**0.030**			0.960			0.374			**0.035**			0.421
T<63	424	181	35.8		84	39.8		31	24.0		4	19.0		31	42.5		24	36.9		15	28.3		4	57.1	
T≥63	581	325	64.2		127	60.2		98	76.0		17	81.0		42	57.5		41	63.1		38	71.7		3	42.9	
**Hostility**				**<0.001**			**0.028**			0.831			0.395			0.088			0.135			0.689			**0.011**
T<63	523	220	43.5		124	58.8		66	51.2		9	42.9		45	61.6		28	43.1		29	54.7		7	100.0	
T≥63	482	286	56.5		87	41.2		63	48.8		12	57.1		28	38.4		37	56.9		24	45.3		0	0.0	
**Phobic Anxiety**				0.105			**0.002**			**<0.001**			0.180			0.074			0.969			0.421			0.315
T<63	528	253	50.0		91	43.1		43	33.3		8	38.1		31	42.5		34	52.3		25	47.2		5	71.4	
T≥63	477	253	50.0		120	56.9		86	66.7		13	61.9		42	57.5		31	47.7		28	52.8		2	28.6	

S4 Table contd.

Diagnosis		Depression			Social phobia			Agoraphbia / Panic disorder			Generalized Anxiety Disorder			Phobia			Somatization			Compulsory disorder			Psychosis		
BSI-scale		n	%	p	n	%	p	n	%	p	n	%	p	n	%	p	n	%	p	n	%	p	n	%	p
**Paranoid Ideation**				**<0.001**			0.278			0.556			0.655			**<0.001**			0.257			0.590			0.606
T<63	623	275	54.3		124	58.8		83	64.3		14	66.7		60	82.2		36	55.4		31	58.5		5	71.4	
T≥63	382	231	45.7		87	41.2		46	35.7		7	33.3		13	17.8		29	44.6		22	41.5		2	28.6	
**Psychoticism**				**<0.001**			0.891			0.631			0.806			**0.005**			0.245			0.901			0.374
T<63	409	141	27.9		85	40.3		55	42.6		8	38.1		41	56.2		22	33.8		22	41.5		4	57.1	
T≥63	596	365	72.1		126	59.7		74	57.4		13	61.9		32	43.8		43	66.2		31	58.5		3	42.9	
**GSI**				**<0.001**			0.146			0.056			0.282			**0.026**			0.230			0.401			0.110
T<63	298	98	19.4		54	25.6		29	22.5		4	19.0		30	41.1		15	23.1		13	24.5		4	57.1	
T≥63	707	408	80.6		157	74.4		100	77.5		17	81.0		43	58.9		50	76.9		40	75.5		3	42.9	
**Overall Caseness**				**<0.001**			0.059			0.394			0.638			**0.015**			0.108			0.332			0.007
No	183	56	11.1		29	13.7		20	15.5		3	14.3		21	28.8		7	10.8		7	13.2		4	57.1	
Case	822	450	88.9		182	86.3		109	84.5		18	85.7		52	71.2		58	89.2		46	86.8		3	42.9	

Abbreviations: BSI Brief Symptom inventory, GSI Global Severity Index

Overall caseness: 2 scales T≥63 or GSI T≥63)

p-values in bold (green) indicate statistical significance

Cells highlighted in yellow indicate the cases where an association is expected between diagnosis and scale caseness (T>63)
