# Supplementary material for: The Brief Symptom Inventory in the Swiss general population: Presentation of norm scores and predictors of psychological distress
Source: PLoS One. 2024 Jul 3;19(7):e0305192. doi: 10.1371/journal.pone.0305192 (PMC11221686; doi:10.1371/journal.pone.0305192)
Supplement: S2 Appendix — (PDF) [file pone.0305192.s004.pdf]

Psychometric properties of the Brief Symptom Inventory in the Swiss general population: Presentation of norm scores and predictors of psychological distress

Gisela Michel [1*], Julia Baenziger [1], Jeannette Brodbeck [2], Luzius Mader [1,3,4], Claudia Kuehni [3,5], Katharina Roser [1]

[1] Faculty of Health Sciences and Medicine, University of Lucerne, Alpenquai 4, 6005 Lucerne, Switzerland; E-mail: gisela.michel@unilu.ch, julia.baenziger@outlook.com, katharina.roser@unilu.ch
[2] Institute of Psychology, University of Bern, Fabrikstrasse 8, 3012 Bern, Switzerland. E-mail: jeannette.brodbeck@unibe.ch
[3] Institute for Social and Preventive Medicine, University of Bern, Mittelstrasse 43, 3012 Bern, Switzerland. E-mail: claudia.kuehni@ispm.unibe.ch
[4] Cancer Registry Bern-Solothurn, University of Bern, Murtenstrasse 31, 3008 Bern, Switzerland. E-mail: luzius.mader@unibe.ch
[5] Pediatric Hematology and Oncology, University Children's Hospital Bern, University of Bern, Freiburgstrasse 15, 3010 Bern, Bern, Switzerland.

*Corresponding author: Gisela Michel, Faculty of Health Sciences and Medicine, University of Lucerne, Alpenquai 4, 6005 Lucerne, Switzerland, E-mail: gisela.michel@unilu.ch

Appendix B

S7 Table: Swiss T-Norms for the Brief Symptom Inventory (BSI; BSI scales and GSI) and the Brief Symptom Inventory-18 (BSI-18; Somatization-6 items and GSI-18)...2
S8 Table: T- standardization of the Brief Symptom Inventory for Switzerland 3
S9 Table: Proportion of clinically relevant cases for each scale and the complete BSI in a sample of the Swiss general population..13

S7 Table: Swiss T-Norms for the Brief Symptom Inventory (BSI; BSI scales and GSI) and the Brief Symptom Inventory-18 (BSI-18; Somatization-6 items and GSI-18)

	T-Score for all			T-Score for males			T-Score for females		
	Mean	95% CI		Mean	95% CI		Mean	95% CI	
**BSI**									
Somatization	50.40	49.87	50.92	50.41	49.62	51.19	50.50	49.80	51.19
Obsessive-compulsive tendencies	50.12	49.57	50.68	50.25	49.39	51.11	50.32	49.60	51.04
Interpersonal sensitivity	50.54	50.03	51.05	50.45	49.68	51.22	50.27	49.56	50.98
Depression	50.45	49.93	50.98	50.73	49.96	51.51	50.59	49.91	51.27
Anxiety	50.48	49.94	51.01	50.54	49.74	51.35	50.21	49.50	50.93
Hostility	50.10	49.56	50.64	50.42	49.61	51.23	50.30	49.61	50.99
Phobic anxiety	50.43	49.96	50.90	51.12	50.42	51.81	50.58	49.96	51.19
Paranoid ideation	50.44	49.91	50.97	50.32	49.49	51.15	50.41	49.73	51.10
Psychoticism	50.65	50.16	51.13	50.91	50.15	51.67	50.64	50.00	51.27
GSI	50.04	49.45	50.62	50.04	49.14	50.95	50.04	49.28	50.80
**BSI-18**									
Somatization (6 items)	51.85	51.30	52.39	50.98	50.16	51.81	52.35	51.62	53.09
GSI-18	50.15	49.59	50.71	50.29	49.42	51.16	50.18	49.44	50.93

Abbreviations: BSI Brief Symptom Inventory; BSI-18 Brief Symptom Inventory 18; CI Confidence Interval; GSI Global Severity Index

S8 Table: T- standardization of the Brief Symptom Inventory for Switzerland

For the standardization either the Sum Score (sum of all items of respective scale) or the Mean score (mean of all items of respective scale) can be used. For each Sum/Mean score of every scale the respective T-score can be found in the table.

		T-Score		
**Sum Score**	**Mean Score**	**All**	**Males**	**Females**
**BSI**				
**Somatization**				
0	0.00	41	42	40
1	0.14	49	51	48
2	0.29	54	56	52
3	0.43	57	59	55
4	0.57	60	62	58
5	0.71	62	64	61
6	0.86	64	66	63
7	1.00	66	68	65
8	1.14	68	70	66
9	1.29	69	72	68
10	1.43	71	73	69
11	1.57	72	75	71
12	1.71	74	76	72
13	1.86	75	76	74
14	2.00	76	77	74
15	2.14	77	79	75
16	2.29	78	79	77
17	2.43	80	79	80
>18	2.57	80	80	80
**Obsessive-compulsive tendencies**				
0	0.00	37	37	37
1	0.17	44	45	44
2	0.33	49	49	48
3	0.50	52	53	52
4	0.67	55	56	55
5	0.83	58	59	58
6	1.00	61	62	60
7	1.17	63	64	63
8	1.33	65	66	64
9	1.50	66	66	65
10	1.67	67	67	66
11	1.83	68	69	68

12	2.00	69	71	69
13	2.17	71	71	70
14	2.33	72	72	71
15	2.50	72	72	73
16	2.67	74	74	74
17	2.83	75	76	75
18	3.00	76	78	75
19	3.17	77	80	76
20	3.33	79	80	77
>21	3.83	80	80	80
**Interpersonal sensitivity**				
0	0.00	42	43	40
1	0.25	50	52	48
2	0.50	55	57	53
3	0.75	58	60	57
4	1.00	61	63	60
5	1.25	64	66	62
6	1.50	66	68	64
7	1.75	67	69	66
8	2.00	69	70	69
9	2.25	71	71	71
10	2.50	72	72	72
11	2.75	73	72	73
12	3.00	74	74	74
13	3.25	76	77	75
14	3.50	78	79	77
15	3.75	80	80	80
>16	4.00	80	80	80
**Depression**				
0	0.00	42	43	42
1	0.17	51	51	50
2	0.33	54	55	54
3	0.50	57	58	57
4	0.67	60	61	59
5	0.83	62	63	61
6	1.00	64	65	63
7	1.17	66	67	65
8	1.33	67	68	66
9	1.50	68	69	67

10	1.67	69	70	68
11	1.83	70	70	70
12	2.00	71	71	70
13	2.17	72	73	72
14	2.33	74	75	74
15	2.50	75	75	76
16	2.67	76	75	77
17	2.83	76	75	79
18	3.00	77	75	80
19	3.17	77	75	80
20	3.33	78	76	80
21	3.50	79	76	80
21	3.67	80	79	80
22	3.67	80	80	80
**Anxiety**				
0	0.00	40	41	39
1	0.17	48	49	47
2	0.33	53	54	52
3	0.50	57	58	55
4	0.67	60	61	58
5	0.83	62	64	61
6	1.00	64	66	63
7	1.17	66	67	65
8	1.33	67	68	67
9	1.50	69	69	68
10	1.67	70	70	69
11	1.83	71	71	71
12	2.00	72	72	72
13	2.17	74	75	73
14	2.33	75	78	74
15	2.50	76	79	75
16	2.67	77	79	75
17	2.83	77	79	76
18	3.00	78	79	77
19	3.17	78	79	78
20	3.33	79	79	79
21	3.50	80	79	80
>22	3.67	80	80	80

**Hostility**				
0	0.00	39	40	39
1	0.20	48	49	48
2	0.40	54	55	53
3	0.60	58	59	57
4	0.80	61	62	60
5	1.00	64	65	63
6	1.20	66	67	65
7	1.40	68	69	67
8	1.60	70	71	70
9	1.80	73	72	73
10	2.00	74	74	75
11	2.20	76	75	76
12	2.40	78	77	78
13	2.60	79	78	80
14	2.80	80	79	80
≥15	3.00	80	80	80
**Phobic anxiety**				
0	0.00	45	46	45
1	0.20	56	57	56
2	0.40	61	62	60
3	0.60	64	66	63
4	0.80	66	67	65
5	1.00	68	69	67
6	1.20	69	70	69
7	1.40	71	72	71
8	1.60	73	72	73
9	1.80	74	73	74
10	2.00	74	73	76
11	2.20	76	75	78
12	2.40	77	75	79
13	2.60	77	76	79
14	2.80	78	76	80
15	3.00	78	77	80
16	3.20	79	78	80
17	3.40	79	79	80
>18	3.60	80	80	80

**Paranoid ideation**				
0	0.00	40	40	40
1	0.20	48	48	47
2	0.40	52	53	52
3	0.60	56	56	55
4	0.80	59	59	59
5	1.00	61	62	61
6	1.20	63	64	63
7	1.40	65	65	65
8	1.60	67	67	66
9	1.80	69	69	68
10	2.00	70	70	70
11	2.20	71	72	71
12	2.40	72	73	72
13	2.60	75	74	75
14	2.80	77	76	80
15	3.00	78	76	80
16	3.20	79	76	80
17	3.40	79	77	80
18	3.60	80	78	80
>19	3.80	80	80	80

**Psychoticism**				
0	0.00	44	44	44
1	0.20	53	54	53
2	0.40	58	59	57
3	0.60	61	63	60
4	0.80	64	66	63
5	1.00	66	67	65
6	1.20	68	68	68
7	1.40	70	69	70
8	1.60	71	70	72
9	1.80	72	72	73
10	2.00	74	74	74
11	2.20	76	76	76
12	2.40	78	76	80
13	2.60	79	77	80
14	2.80	80	78	80
15	3.00	80	78	80
16	3.20	80	79	80
>17	3.40	80	80	80

GSI				
0	0.00	30	30	29
1	0.02	34	35	34
2	0.04	37	38	36
3	0.06	39	40	38
4	0.08	40	41	39
5	0.09	42	43	41
6	0.11	43	44	42
7	0.13	45	46	44
8	0.15	46	47	44
9	0.17	47	48	45
10	0.19	47	49	46
11	0.21	48	50	47
12	0.23	49	50	48
13	0.25	50	51	49
14	0.26	51	52	49
15	0.28	51	53	50
16	0.30	52	54	51
17	0.32	53	54	51
18	0.34	53	55	52
19	0.36	54	56	52
20	0.38	54	56	53
21	0.40	55	56	54
22	0.42	55	57	54
23	0.43	56	58	55
24	0.45	57	58	55
25	0.47	57	59	56
26	0.49	57	59	56
27	0.51	58	59	56
28	0.53	58	60	57
29	0.55	59	60	57
30	0.57	59	61	58
31	0.58	60	61	58
32	0.60	60	62	59
33	0.62	61	62	59
34	0.64	61	62	60
35	0.66	61	63	60
36	0.68	62	63	61
37	0.70	62	63	61
38	0.72	62	64	62
39	0.74	63	64	62

40	0.75	63	64	62
41	0.77	63	65	62
42	0.79	63	65	62
43	0.81	64	65	63
44	0.83	64	65	63
45	0.85	64	66	63
46	0.87	64	66	63
47	0.89	65	67	63
48	0.91	65	67	64
49	0.92	65	67	64
50	0.95	65	67	64
51	0.96	65	67	64
52	0.98	66	67	64
53	1.00	66	67	65
54	1.02	66	67	65
55	1.04	66	68	65
56	1.06	66	68	65
57	1.08	66	68	65
58	1.09	66	68	65
59	1.11	67	68	65
60	1.13	67	68	66
61	1.15	67	68	66
62	1.17	67	68	66
63	1.19	67	68	66
64	1.21	68	69	67
65	1.23	68	69	67
66	1.25	68	69	67
67	1.27	68	69	67
68	1.28	68	69	67
69	1.30	68	69	68
70	1.32	69	69	68
71	1.34	69	69	68
70	1.32	69	69	68
73	1.38	69	69	68
74	1.40	69	69	69
75	1.42	69	70	69
76	1.43	69	70	69
77	1.46	69	70	69
78	1.47	69	70	69
79	1.49	70	70	70
80	1.51	70	70	70

81	1.53	71	70	71
82	1.55	71	70	71
83	1.57	71	71	71
84	1.58	71	71	71
85	1.60	71	71	72
86	1.62	72	71	73
87	1.64	72	71	73
88	1.66	72	71	73
89	1.68	72	71	73
90	1.70	72	71	74
91	1.72	73	71	74
92	1.74	73	72	75
93	1.76	73	72	75
94	1.78	73	72	75
95	1.80	73	72	75
96	1.81	73	72	75
97	1.83	74	72	75
98	1.85	74	72	76
99	1.87	74	72	76
100	1.89	74	72	76
101	1.91	74	72	76
102	1.93	74	72	76
103	1.94	74	72	76
104	1.96	75	73	77
105	1.98	76	74	77
106	2.00	76	75	78
107	2.02	76	75	78
108	2.04	76	75	78
109	2.06	76	75	78
110	2.08	77	76	79
111	2.10	77	76	79
112	2.11	77	76	79
113	2.14	77	76	79
114	2.16	78	76	80
115	2.17	78	76	80
116	2.19	78	76	80
117	2.21	78	76	80
118	2.23	79	77	80
119	2.25	80	78	80
120	2.27	80	79	80
>121	2.29	80	80	80

**BSI-18**				
**Somatization (6 items)**				
0	0.00	42	42	41
1	0.17	51	52	50
2	0.33	55	57	54
3	0.50	59	60	58
4	0.67	61	63	60
5	0.83	64	65	63
6	1.00	66	67	65
7	1.17	68	69	67
8	1.33	70	71	69
9	1.50	72	74	71
10	1.67	74	74	73
11	1.83	75	75	74
12	2.00	76	77	75
13	2.17	78	79	78
>14	2.33	80	80	80

**GSI-18**				
0	0.00	36	36	35
1	0.06	42	42	42
2	0.11	46	47	45
3	0.17	48	50	47
4	0.22	50	52	49
5	0.28	52	54	51
6	0.33	54	56	53
7	0.39	55	57	54
8	0.44	57	59	56
9	0.50	59	60	57
10	0.56	60	61	59
11	0.61	61	62	60
12	0.67	62	63	61
13	0.72	63	64	62
14	0.78	63	65	62
15	0.83	64	66	63
16	0.89	65	66	64
17	0.94	65	67	64
18	1.00	66	67	65
19	1.06	66	68	65

20	1.11	67	69	65
21	1.17	67	70	66
22	1.22	68	70	66
23	1.28	68	70	67
24	1.33	69	70	68
25	1.39	69	71	69
26	1.44	70	71	69
27	1.50	70	71	70
28	1.56	71	71	71
29	1.61	72	71	72
30	1.67	72	72	72
31	1.72	73	72	74
32	1.78	73	72	75
33	1.83	74	72	76
34	1.89	74	73	76
35	1.94	75	73	76
36	2.00	75	74	76
37	2.06	76	75	77
38	2.11	76	75	77
39	2.17	77	76	78
40	2.22	79	78	79
41	2.28	79	78	79
42	2.33	80	79	80
43	2.39	80	79	80
44	2.44	80	79	80
45	2.50	80	79	80
46	2.56	80	79	80
>47	2.61	80	80	80

Abbreviations: BSI Brief Symptom Inventory, BSI-18 Brief Symptom Inventory 18, GSI Global Severity Index, GSI-18 Global Severity Index for the Brief Symptom Inventory 18 (only including items of the Somatization (6 items), Depression and Anxiety scale

S9 Table: Proportion of clinically relevant cases for each scale and the complete BSI in a sample of the Swiss general population

	All			Male			Female		
	%	95% CI		%	95% CI		%	95% CI	
**Somatization**									
No distress	90.8	89.1	92.4	90.5	87.6	92.8	88.4	85.8	90.6
Distress	9.2	7.6	10.9	9.5	7.2	12.4	11.6	9.4	14.2
**Obsessive-compulsive tendencies**									
No distress	89.3	87.2	91.0	90.2	87.1	92.7	88.3	85.6	90.6
Distress	10.7	9.0	12.8	9.8	7.3	12.9	11.7	9.4	14.4
**Interpersonal sensitivity**									
No distress	89.7	87.7	91.4	88.0	84.6	90.7	90.9	88.4	93.0
Distress	10.3	8.6	12.3	12.0	9.3	15.4	9.1	7.0	11.6
**Depression**									
No distress	90.2	88.3	91.8	88.9	85.6	91.5	89.1	86.4	91.4
Distress	9.8	8.2	11.7	11.1	8.5	14.4	10.9	8.6	13.6
**Anxiety**									
No distress	91.1	89.3	92.7	89.9	86.6	92.4	88.8	86.1	91.1
Distress	8.9	7.3	10.7	10.1	7.6	13.4	11.2	8.9	13.9
**Hostility**									
No distress	89.8	87.8	91.4	90.9	88.0	93.2	88.7	86.0	90.9
Distress	10.2	8.6	12.2	9.1	6.8	12.0	11.3	9.1	14.0
**Phobic anxiety**									
No distress	90.0	88.1	91.6	92.9	90.3	94.8	87.3	84.5	89.6
Distress	10.0	8.4	11.9	7.1	5.2	9.7	12.7	10.4	15.5
**Paranoid ideation**									
No distress	90.0	88.1	91.7	90.5	87.4	92.8	89.6	87.0	91.8
Distress	10.0	8.3	11.9	9.5	7.2	12.6	10.4	8.2	13.0
**Psychoticism**									
No distress	90.3	88.3	91.9	86.7	83.3	89.6	87.9	85.1	90.3
Distress	9.7	8.1	11.7	13.3	10.4	16.7	12.1	9.7	14.9
**GSI**									
No distress	89.8	87.9	91.4	89.4	86.3	91.9	89.5	86.9	91.7
Distress	10.2	8.6	12.1	10.6	8.1	13.7	10.5	8.3	13.1
**Case with Distress (T≥63 on at least 2 scales, or T≥63 on the GSI)**									
No distress	81.9	79.5	84.1	81.3	77.5	84.6	79.3	76.0	82.3
Distress	18.1	16.0	20.5	18.7	15.4	22.5	20.7	17.7	24.0

Abbreviations: BSI Brief Symptom Inventory
