# Supplementary material for: The Brief Symptom Inventory in the Swiss general population: Presentation of norm scores and predictors of psychological distress
Source: PLoS One. 2024 Jul 3;19(7):e0305192. doi: 10.1371/journal.pone.0305192 (PMC11221686; doi:10.1371/journal.pone.0305192)
Supplement: S3 Appendix — (PDF) [file pone.0305192.s005.pdf]

Psychometric properties of the Brief Symptom Inventory in the Swiss general population: Presentation of norm scores and predictors of psychological distress

Gisela Michel [1*], Julia Baenziger [1], Jeannette Brodbeck [2], Luzius Mader [1,3,4], Claudia Kuehni [3,5], Katharina Roser [1]

[1] Faculty of Health Sciences and Medicine, University of Lucerne, Alpenquai 4, 6005 Lucerne, Switzerland; E-mail: gisela.michel@unilu.ch, julia.baenziger@outlook.com, katharina.roser@unilu.ch
[2] Institute of Psychology, University of Bern, Fabrikstrasse 8, 3012 Bern, Switzerland. E-mail: jeannette.brodbeck@unibe.ch
[3] Institute for Social and Preventive Medicine, University of Bern, Mittelstrasse 43, 3012 Bern, Switzerland. E-mail: claudia.kuehni@ispm.unibe.ch
[4] Cancer Registry Bern-Solothurn, University of Bern, Murtenstrasse 31, 3008 Bern, Switzerland. E-mail: luzius.mader@unibe.ch
[5] Pediatric Hematology and Oncology, University Children's Hospital Bern, University of Bern, Freiburgstrasse 15, 3010 Bern, Bern, Switzerland.

*Corresponding author: Gisela Michel, Faculty of Health Sciences and Medicine, University of Lucerne, Alpenquai 4, 6005 Lucerne, Switzerland, E-mail: gisela.michel@unilu.ch

Appendix C

S10 Tables: Descriptive statistics for the scales and the GSI of the BSI for the representative Swiss population sample (Sum score, Mean score, T-Scores (Standardization according to German and Swiss norms), Positive Symptom Total; all based on weighted analyses)

Content:

a) Total sample of the Swiss general population 2
b) Total sample of the Swiss general population by sex 3
c) Total sample of the Swiss general population by 10-year age groups 5
d) Total sample of the Swiss general population by language 8
e) Total sample of the Swiss general population by education 10
f) Total sample of the Swiss general population by employment 13
g) Total sample of the Swiss general population by migration background 15

Abbreviations: BSI Brief Symptom Inventory, BSI-18 Brief Symptom Inventory 18, GSI Global Severity Index, GSI-18 Global Severity Index for the Brief Symptom Inventory 18 (only including items of the Somatization (6 items), Depression and Anxiety scale

Sum Score: sum of all items of respective scale; Mean score: mean of all items of respective scale

a) Total sample of the Swiss general population

(corresponds to Table 4 in manuscript, added here for completeness)

	**Sum Score**			**Mean Score**			**BSI Positive Symptom Total (PST)**			**T-Standardization (German norms)**			**T-Standardization (Swiss norms)**		
	**Mean**	**95% CI**		**Mean**	**95% CI**		**Mean**	**95% CI**		**Mean**	**95% CI**		**Mean**	**95% CI**	
Somatization	1.97	1.82	2.12	0.28	0.26	0.30	1.57	1.47	1.66	50.2	49.7	50.8	50.4	49.9	50.9
Obsessive-compulsive tendencies	3.12	2.93	3.31	0.52	0.49	0.55	2.46	2.35	2.57	49.6	49.0	50.2	50.1	49.6	50.7
Interpersonal sensitivity	1.71	1.57	1.84	0.43	0.39	0.46	1.28	1.20	1.36	49.7	49.1	50.3	50.5	50.0	51.0
Depression	1.94	1.76	2.12	0.32	0.29	0.35	1.45	1.35	1.54	50.5	49.9	51.1	50.5	49.9	51.0
Anxiety	2.15	1.99	2.31	0.36	0.33	0.38	1.60	1.51	1.69	50.1	49.5	50.7	50.5	49.9	51.0
Hostility	1.83	1.70	1.95	0.37	0.34	0.39	1.37	1.30	1.45	50.4	49.8	51.0	50.1	49.6	50.6
Phobic anxiety	0.85	0.74	0.95	0.17	0.15	0.19	0.62	0.56	0.68	50.6	50.1	51.1	50.4	50.0	50.9
Paranoid ideation	2.25	2.09	2.41	0.45	0.42	0.48	1.71	1.62	1.80	52.3	51.7	52.9	50.4	49.9	51.0
Psychoticism	1.18	1.06	1.30	0.24	0.21	0.26	0.88	0.81	0.95	51.5	51.0	52.1	50.6	50.2	51.1
GSI	18.57	17.44	19.70	0.35	0.33	0.37	14.14	13.54	14.73	49.8	49.0	50.5	50.0	49.5	50.6
**BSI-18**							**Positive Symptom Distress Index (PSDI)**								
Somatization (6 items)	1.63	1.50	1.75	0.27	0.25	0.29	1.24	1.22	1.26				51.8	51.3	52.4
GSI-18 (accepted number of missings ≤2)	5.71	5.32	6.10	0.32	0.30	0.34							50.2	49.6	50.7

b) Total sample of the Swiss general population by sex

	Sum Score			Mean Score			BSI Positive Symptom Total (PST)			T-Standardization (German norms)			T-Standardization (Swiss norms)		
	**Mean**	**95% CI**		**Mean**	**95% CI**		**Mean**	**95% CI**		**Mean**	**95% CI**		**Mean**	**95% CI**	
Somatization															
Male	1.54	1.34	1.74	0.22	0.19	0.25	1.27	1.14	1.41	48.6	47.7	49.4	48.8	48.1	49.6
Female	2.37	2.16	2.58	0.34	0.31	0.37	1.84	1.70	1.97	51.8	51.1	52.6	51.9	51.2	52.6
Obsessive-compulsive tendencies															
Male	2.95	2.67	3.23	0.49	0.45	0.54	2.39	2.22	2.55	49.0	48.1	50.0	49.6	48.8	50.5
Female	3.28	3.02	3.54	0.55	0.50	0.59	2.53	2.39	2.67	50.1	49.3	50.9	50.6	49.9	51.3
Interpersonal sensitivity															
Male	1.35	1.15	1.55	0.34	0.29	0.39	1.02	0.91	1.14	47.8	46.9	48.7	49.0	48.2	49.7
Female	2.04	1.85	2.23	0.51	0.46	0.56	1.52	1.41	1.62	51.4	50.6	52.3	52.0	51.3	52.7
Depression															
Male	1.79	1.53	2.05	0.30	0.26	0.34	1.35	1.21	1.50	49.9	49.0	50.8	50.0	49.2	50.7
Female	2.08	1.84	2.32	0.35	0.31	0.39	1.53	1.40	1.66	51.1	50.3	51.9	50.9	50.2	51.6
Anxiety															
Male	1.87	1.64	2.10	0.31	0.27	0.35	1.43	1.30	1.56	49.0	48.0	49.9	49.5	48.7	50.3
Female	2.40	2.18	2.62	0.40	0.36	0.44	1.75	1.63	1.87	51.2	50.3	52.0	51.4	50.7	52.1
Hostility															
Male	1.70	1.51	1.88	0.34	0.30	0.38	1.32	1.21	1.43	49.7	48.8	50.6	49.5	48.6	50.3
Female	1.95	1.79	2.12	0.39	0.36	0.42	1.42	1.33	1.52	51.1	50.3	51.9	50.7	50.0	51.4
Phobic anxiety															
Male	0.78	0.62	0.95	0.16	0.12	0.19	0.56	0.48	0.65	50.2	49.4	50.9	50.1	49.4	50.8
Female	0.91	0.77	1.04	0.18	0.15	0.21	0.68	0.59	0.76	51.0	50.3	51.7	50.7	50.1	51.4
Paranoid ideation															
Male	2.17	1.93	2.42	0.43	0.39	0.48	1.63	1.49	1.77	52.0	51.1	52.9	50.1	49.3	50.9
Female	2.32	2.11	2.52	0.46	0.42	0.50	1.78	1.66	1.90	52.7	51.9	53.4	50.8	50.1	51.4
Psychoticism															
Male	1.11	0.93	1.28	0.22	0.19	0.26	0.85	0.75	0.95	51.2	50.4	52.0	50.4	49.7	51.1
Female	1.25	1.09	1.41	0.25	0.22	0.28	0.91	0.82	1.01	51.8	51.1	52.6	50.9	50.2	51.5

GSI															
Male	16.61	14.91	18.31	0.31	0.28	0.35	12.89	11.99	13.79	48.2	47.1	49.3	48.9	48.0	49.7
Female	20.41	18.92	21.90	0.39	0.36	0.41	15.29	14.51	16.08	51.3	50.3	52.3	51.1	50.4	51.9
**BSI-18**															
Somatization (6 items)								**Positive Symptom Distress Index (PSDI)**							
Male	1.41	1.22	1.59	0.23	0.20	0.26	1.21	1.18	1.24				50.2	49.5	51.0
Female	1.83	1.65	2.01	0.31	0.28	0.33	1.26	1.23	1.29				53.3	52.6	54.1
GSI-18 (accepted number of missings ≤2)															
Male	5.07	4.50	5.65	0.28	0.25	0.31							49.2	48.4	50.0
Female	6.31	5.78	6.84	0.35	0.32	0.38							51.1	50.3	51.8

c) Total sample of the Swiss general population by 10-year age groups

	**Sum Score**			**Mean Score**			**BSI Positive Symptom Total (PST)**			**T-Standardization (German norms)**			**T-Standardization (Swiss norms)**		
	**Mean**	**95% CI**		**Mean**	**95% CI**		**Mean**	**95% CI**		**Mean**	**95% CI**		**Mean**	**95% CI**	
Somatization															
18-25 years	2.38	1.76	3.00	0.34	0.25	0.43	1.78	1.38	2.18	51.5	49.3	53.7	51.6	49.5	53.7
26-35 years	1.41	1.09	1.72	0.20	0.16	0.25	1.11	0.90	1.33	48.0	46.6	49.3	48.2	47.0	49.5
36-45 years	1.59	1.28	1.90	0.23	0.18	0.27	1.29	1.08	1.51	48.5	47.3	49.8	48.8	47.7	50.0
46-55 years	2.02	1.73	2.31	0.29	0.25	0.33	1.66	1.46	1.86	50.8	49.7	51.9	50.9	49.9	51.9
56-65 years	2.26	1.86	2.66	0.32	0.27	0.38	1.80	1.55	2.04	51.3	50.0	52.7	51.4	50.1	52.6
66-75 years	2.44	2.06	2.81	0.35	0.30	0.40	1.90	1.66	2.14	52.3	50.9	53.6	52.3	51.0	53.5
Obsessive-compulsive tendencies															
18-25 years	4.40	3.55	5.25	0.73	0.59	0.87	2.97	2.59	3.35	53.5	50.9	56.0	53.4	51.2	55.6
26-35 years	3.12	2.63	3.61	0.52	0.44	0.60	2.29	2.01	2.57	49.6	47.9	51.2	50.1	48.6	51.6
36-45 years	3.00	2.58	3.43	0.50	0.43	0.57	2.45	2.22	2.68	49.4	48.0	50.7	50.0	48.8	51.2
46-55 years	3.00	2.59	3.41	0.50	0.43	0.57	2.35	2.12	2.57	48.7	47.4	50.1	49.4	48.2	50.6
56-65 years	2.89	2.50	3.28	0.48	0.42	0.55	2.49	2.23	2.75	49.0	47.6	50.4	49.6	48.4	50.8
66-75 years	2.92	2.52	3.32	0.49	0.42	0.55	2.51	2.26	2.76	49.4	48.0	50.8	50.0	48.8	51.2
Interpersonal sensitivity															
18-25 years	2.73	2.07	3.39	0.68	0.52	0.85	1.73	1.42	2.05	53.8	51.1	56.6	53.7	51.5	55.8
26-35 years	1.82	1.43	2.21	0.45	0.36	0.55	1.26	1.05	1.47	50.0	48.4	51.7	50.9	49.5	52.3
36-45 years	1.71	1.40	2.02	0.43	0.35	0.50	1.31	1.12	1.49	49.9	48.5	51.3	50.7	49.5	51.9
46-55 years	1.48	1.21	1.76	0.37	0.30	0.44	1.13	0.96	1.29	48.3	47.1	49.5	49.4	48.4	50.4
56-65 years	1.51	1.25	1.78	0.38	0.31	0.44	1.28	1.10	1.46	49.0	47.8	50.3	50.0	49.0	51.1
66-75 years	1.49	1.24	1.75	0.37	0.31	0.44	1.23	1.05	1.40	49.2	47.9	50.5	50.2	49.1	51.3
Depression															
18-25 years	2.94	2.18	3.70	0.49	0.36	0.62	1.96	1.58	2.34	53.9	51.4	56.3	53.3	51.2	55.3
26-35 years	2.43	1.88	2.97	0.40	0.31	0.49	1.67	1.39	1.95	52.0	50.2	53.7	51.8	50.2	53.3
36-45 years	1.87	1.50	2.24	0.31	0.25	0.37	1.46	1.23	1.68	50.5	49.1	51.8	50.4	49.2	51.6
46-55 years	1.73	1.39	2.07	0.29	0.23	0.35	1.30	1.12	1.49	49.5	48.4	50.7	49.6	48.6	50.6
56-65 years	1.57	1.23	1.91	0.26	0.21	0.32	1.28	1.07	1.50	49.3	48.1	50.5	49.5	48.4	50.5
66-75 years	1.59	1.24	1.93	0.26	0.21	0.32	1.26	1.06	1.47	49.5	48.2	50.8	49.7	48.5	50.8
Anxiety															
18-25 years	2.95	2.32	3.57	0.49	0.39	0.60	2.12	1.81	2.43	53.5	51.2	55.9	53.5	51.5	55.4
26-35 years	2.49	2.08	2.89	0.41	0.35	0.48	1.79	1.56	2.02	52.1	50.5	53.7	52.2	50.8	53.6
36-45 years	2.22	1.86	2.58	0.37	0.31	0.43	1.67	1.47	1.87	50.7	49.2	52.2	50.9	49.7	52.2

46-55 years	1.99	1.65	2.33	0.33	0.27	0.39	1.42	1.25	1.58	49.2	47.9	50.4	49.7	48.6	50.8
56-65 years	2.04	1.67	2.42	0.34	0.28	0.40	1.58	1.36	1.81	49.4	47.8	50.9	49.8	48.5	51.0
66-75 years	1.52	1.21	1.83	0.25	0.20	0.31	1.26	1.06	1.46	47.2	45.8	48.6	48.0	46.8	49.2
Hostility															
18-25 years	2.54	2.04	3.05	0.51	0.41	0.61	1.76	1.49	2.03	53.8	51.4	56.1	53.1	51.0	55.2
26-35 years	1.93	1.60	2.25	0.39	0.32	0.45	1.39	1.19	1.59	51.0	49.4	52.6	50.6	49.2	52.1
36-45 years	2.04	1.76	2.33	0.41	0.35	0.47	1.56	1.38	1.74	51.8	50.3	53.2	51.3	50.0	52.6
46-55 years	1.66	1.40	1.91	0.33	0.28	0.38	1.24	1.11	1.37	49.5	48.3	50.7	49.3	48.2	50.3
56-65 years	1.61	1.34	1.88	0.32	0.27	0.37	1.29	1.13	1.46	49.3	47.9	50.6	49.0	47.8	50.2
66-75 years	1.52	1.23	1.81	0.30	0.25	0.36	1.19	1.03	1.36	48.8	47.4	50.2	48.6	47.4	49.9
Phobic anxiety															
18-25 years	1.49	0.98	1.99	0.30	0.20	0.40	0.96	0.68	1.24	53.6	51.2	56.0	52.8	50.8	54.9
26-35 years	0.83	0.50	1.15	0.16	0.10	0.23	0.53	0.38	0.69	50.1	48.8	51.4	50.0	48.8	51.2
36-45 years	0.70	0.51	0.88	0.14	0.10	0.18	0.53	0.41	0.66	50.0	48.8	51.1	49.8	48.8	50.8
46-55 years	0.67	0.48	0.87	0.13	0.10	0.17	0.48	0.38	0.58	49.5	48.6	50.4	49.5	48.6	50.3
56-65 years	0.96	0.71	1.21	0.19	0.14	0.24	0.76	0.60	0.92	51.4	50.2	52.6	51.2	50.1	52.3
66-75 years	0.80	0.61	0.99	0.16	0.12	0.20	0.68	0.53	0.84	50.9	49.8	52.1	50.8	49.8	51.8
Paranoid ideation															
18-25 years	2.94	2.29	3.59	0.59	0.46	0.72	2.02	1.69	2.36	54.6	52.4	56.9	52.5	50.5	54.6
26-35 years	2.21	1.82	2.60	0.44	0.36	0.52	1.66	1.41	1.90	52.3	50.7	53.9	50.3	48.9	51.7
36-45 years	2.18	1.78	2.58	0.44	0.36	0.52	1.61	1.39	1.83	51.8	50.3	53.2	49.9	48.6	51.2
46-55 years	2.20	1.87	2.53	0.44	0.37	0.51	1.64	1.46	1.82	52.1	50.9	53.3	50.2	49.1	51.3
56-65 years	2.43	2.06	2.80	0.49	0.41	0.56	1.95	1.73	2.17	53.2	51.8	54.5	51.3	50.1	52.5
66-75 years	1.82	1.55	2.09	0.36	0.31	0.42	1.53	1.33	1.73	51.2	49.9	52.4	49.4	48.3	50.5
Psychoticism															
18-25 years	2.02	1.52	2.52	0.40	0.30	0.50	1.30	1.04	1.56	55.7	53.4	58.1	54.2	52.2	56.2
26-35 years	1.25	0.95	1.55	0.25	0.19	0.31	0.90	0.71	1.08	51.8	50.3	53.4	50.9	49.5	52.2
36-45 years	1.18	0.90	1.46	0.24	0.18	0.29	0.92	0.75	1.09	51.5	50.2	52.8	50.6	49.5	51.7
46-55 years	1.06	0.81	1.31	0.21	0.16	0.26	0.76	0.63	0.90	50.6	49.5	51.6	49.8	48.9	50.8
56-65 years	1.02	0.78	1.26	0.21	0.16	0.25	0.85	0.70	1.01	51.0	49.8	52.1	50.2	49.2	51.2
66-75 years	0.96	0.73	1.19	0.19	0.15	0.24	0.78	0.64	0.92	50.8	49.6	51.9	50.0	49.0	51.1
GSI															
18-25 years	26.33	21.39	31.26	0.50	0.40	0.59	17.95	15.60	20.31	54.4	51.2	57.6	53.4	51.0	55.7
26-35 years	19.03	16.15	21.92	0.36	0.30	0.41	13.74	12.21	15.27	50.3	48.4	52.3	50.4	48.9	51.9
36-45 years	17.89	15.48	20.30	0.34	0.29	0.38	13.92	12.58	15.25	49.4	47.7	51.2	49.7	48.4	51.1
46-55 years	17.32	14.92	19.71	0.33	0.28	0.37	13.09	11.94	14.24	48.5	47.1	50.0	49.1	48.0	50.3
56-65 years	17.88	15.25	20.51	0.34	0.29	0.39	14.55	13.07	16.03	49.1	47.4	50.8	49.6	48.2	51.0

66-75 years	16.80	14.55	19.05	0.32	0.27	0.36	13.68	12.30	15.06	49.4	47.8	51.0	49.8	48.5	51.0
**BSI-18**															
Somatization (6 items)							**Positive Symptom Distress Index (PSDI)**								
18-25 years	1.97	1.44	2.51	0.33	0.24	0.42	1.40	1.32	1.48				53.0	50.9	55.1
26-35 years	1.24	0.96	1.51	0.21	0.16	0.25	1.28	1.23	1.33				49.7	48.4	51.0
36-45 years	1.38	1.11	1.65	0.23	0.18	0.27	1.22	1.18	1.27				50.2	49.0	51.4
46-55 years	1.60	1.36	1.85	0.27	0.23	0.31	1.22	1.18	1.27				52.4	51.3	53.5
56-65 years	1.78	1.44	2.12	0.30	0.24	0.35	1.18	1.14	1.22				52.9	51.6	54.2
66-75 years	2.04	1.70	2.37	0.34	0.28	0.39	1.19	1.15	1.23				53.8	52.5	55.1
GSI-18 (accepted number of missings ≤2)															
18-25 years	7.86	6.26	9.46	0.44	0.35	0.53							53.2	51.1	55.3
26-35 years	6.15	5.12	7.18	0.34	0.28	0.40							51.0	49.6	52.5
36-45 years	5.47	4.63	6.32	0.30	0.26	0.35							49.8	48.5	51.1
46-55 years	5.32	4.52	6.11	0.30	0.25	0.34							49.4	48.3	50.5
56-65 years	5.39	4.47	6.31	0.30	0.25	0.35							49.5	48.2	50.8
66-75 years	5.14	4.28	6.01	0.29	0.24	0.33							49.7	48.4	50.9

d) ***Total sample of the Swiss general population by language***

(German, French, Italian)

	Sum Score			Mean Score			BSI Positive Symptom Total (PST)			T-Standardization (German norms)			T-Standardization (Swiss norms)		
	**Mean**	**95% CI**		**Mean**	**95% CI**		**Mean**	**95% CI**		**Mean**	**95% CI**		**Mean**	**95% CI**	
Somatization															
German	1.91	1.75	2.08	0.27	0.25	0.30	1.53	1.42	1.65	50.1	49.5	50.8	50.3	49.7	50.9
French	1.79	1.48	2.10	0.25	0.21	0.30	1.46	1.24	1.67	49.4	48.2	50.7	49.6	48.5	50.7
Italian	3.39	2.47	4.31	0.48	0.35	0.62	2.35	1.83	2.88	55.2	52.3	58.1	55.0	52.3	57.6
Obsessive-compulsive tendencies															
German	3.15	2.93	3.37	0.53	0.49	0.56	2.51	2.39	2.63	49.8	49.1	50.5	50.3	49.7	51.0
French	2.85	2.42	3.27	0.47	0.40	0.54	2.28	2.04	2.53	48.3	46.9	49.7	49.0	47.8	50.3
Italian	3.78	2.79	4.77	0.63	0.47	0.80	2.50	1.98	3.03	51.4	48.2	54.6	51.5	48.8	54.3
Interpersonal sensitivity															
German	1.73	1.58	1.89	0.43	0.39	0.47	1.34	1.24	1.43	50.0	49.3	50.7	50.9	50.3	51.4
French	1.46	1.15	1.76	0.37	0.29	0.44	1.06	0.88	1.24	48.0	46.6	49.4	49.0	47.9	50.2
Italian	2.30	1.56	3.03	0.57	0.39	0.76	1.39	1.04	1.74	51.9	48.8	55.0	52.3	49.8	54.8
Depression															
German	1.98	1.77	2.18	0.33	0.30	0.36	1.51	1.39	1.62	50.7	50.0	51.4	50.6	50.0	51.2
French	1.53	1.18	1.88	0.25	0.20	0.31	1.11	0.93	1.30	48.9	47.6	50.1	49.1	48.0	50.2
Italian	3.08	2.08	4.07	0.51	0.35	0.68	1.92	1.46	2.37	54.1	51.3	57.0	53.6	51.1	56.1
Anxiety															
German	1.93	1.76	2.10	0.32	0.29	0.35	1.52	1.42	1.62	49.3	48.6	50.0	49.8	49.2	50.4
French	2.46	2.06	2.87	0.41	0.34	0.48	1.68	1.47	1.88	51.2	49.7	52.7	51.2	50.0	52.5
Italian	3.68	2.80	4.56	0.61	0.47	0.76	2.29	1.87	2.71	56.4	53.7	59.1	55.8	53.5	58.1
Hostility															
German	1.76	1.63	1.89	0.35	0.33	0.38	1.36	1.28	1.44	50.4	49.7	51.0	50.0	49.4	50.6
French	1.81	1.49	2.12	0.36	0.30	0.42	1.28	1.11	1.46	49.6	48.1	51.0	49.3	48.0	50.6
Italian	2.84	2.10	3.58	0.57	0.42	0.72	1.87	1.46	2.29	54.9	51.7	58.1	54.0	51.2	56.8
Phobic anxiety															
German	0.86	0.74	0.98	0.17	0.15	0.20	0.64	0.57	0.71	50.8	50.2	51.4	50.7	50.2	51.2
French	0.80	0.57	1.03	0.16	0.11	0.21	0.58	0.44	0.73	50.2	49.0	51.4	49.8	48.8	50.9
Italian	0.84	0.27	1.42	0.17	0.05	0.28	0.56	0.23	0.89	49.8	47.3	52.3	49.3	47.1	51.5

Paranoid ideation															
German	2.12	1.95	2.29	0.42	0.39	0.46	1.67	1.56	1.77	52.0	51.3	52.7	50.1	49.5	50.7
French	2.44	2.05	2.83	0.49	0.41	0.57	1.72	1.52	1.92	52.7	51.3	54.0	50.8	49.6	52.0
Italian	3.18	2.35	4.00	0.63	0.47	0.80	2.13	1.69	2.56	55.8	53.0	58.5	53.6	51.1	56.0
Psychoticism															
German	1.15	1.02	1.28	0.23	0.20	0.26	0.88	0.80	0.95	51.5	50.9	52.2	50.7	50.1	51.2
French	1.15	0.88	1.42	0.23	0.18	0.28	0.85	0.69	1.01	51.1	49.8	52.4	50.2	49.1	51.3
Italian	1.71	0.99	2.42	0.34	0.20	0.48	1.06	0.69	1.42	53.2	50.3	56.1	52.0	49.6	54.5
GSI															
German	18.22	16.98	19.45	0.34	0.32	0.37	14.22	13.55	14.88	49.8	49.0	50.7	50.1	49.4	50.7
French	17.60	15.05	20.15	0.33	0.28	0.38	12.95	11.61	14.30	48.4	46.6	50.2	48.9	47.5	50.3
Italian	26.96	20.02	33.89	0.51	0.38	0.64	17.47	14.16	20.79	54.5	50.9	58.0	53.8	50.9	56.7
**BSI-18**															
Somatization (6 items)								**Positive Symptom Distress Index (PSDI)**							
German	1.57	1.42	1.71	0.26	0.24	0.28	1.21	1.19	1.23				51.7	51.1	52.3
French	1.50	1.24	1.77	0.25	0.21	0.29	1.29	1.24	1.34				51.0	49.8	52.2
Italian	2.87	2.09	3.65	0.48	0.35	0.61	1.36	1.25	1.47				56.7	53.9	59.5
GSI-18 (accepted number of missings ≤2)															
German	5.47	5.05	5.90	0.30	0.28	0.33							49.9	49.3	50.6
French	5.50	4.61	6.38	0.31	0.26	0.35							49.6	48.4	50.8
Italian	9.63	7.22	12.03	0.53	0.40	0.67							55.3	52.7	58.0

e) Total sample of the Swiss general population by education

(University only includes those with at least Master or higher degree from University, Bachelor from University is coded as upper secondary education)

	Sum Score			Mean Score			BSI Positive Symptom Total (PST)			T-Standardization (German norms)			T-Standardization (Swiss norms)		
	**Mean**	**95% CI**		**Mean**	**95% CI**		**Mean**	**95% CI**		**Mean**	**95% CI**		**Mean**	**95% CI**	
Somatization															
Compulsory schooling	2.86	2.17	3.55	0.41	0.31	0.51	2.12	1.68	2.55	53.5	51.3	55.6	53.4	51.4	55.4
Vocational training	2.22	1.98	2.45	0.32	0.28	0.35	1.73	1.58	1.88	51.2	50.4	52.1	51.3	50.5	52.1
Upper secondary education	1.57	1.33	1.80	0.22	0.19	0.26	1.34	1.17	1.51	49.0	48.0	50.0	49.2	48.3	50.1
University education	1.29	1.05	1.53	0.18	0.15	0.22	1.12	0.92	1.32	47.7	46.5	48.9	48.0	46.9	49.2
Obsessive-compulsive tendencies															
Compulsory schooling	4.15	3.28	5.03	0.69	0.55	0.84	2.85	2.44	3.26	52.6	50.0	55.1	52.6	50.4	54.8
Vocational training	3.30	3.02	3.59	0.55	0.50	0.60	2.62	2.46	2.79	50.2	49.2	51.1	50.6	49.8	51.5
Upper secondary education	2.54	2.24	2.85	0.42	0.37	0.47	2.16	1.96	2.35	47.9	46.8	49.0	48.7	47.7	49.6
University education	2.87	2.43	3.30	0.48	0.41	0.55	2.27	2.02	2.51	48.8	47.4	50.2	49.5	48.2	50.8
Interpersonal sensitivity															
Compulsory schooling	2.43	1.78	3.08	0.61	0.44	0.77	1.54	1.23	1.86	52.4	49.7	55.1	52.6	50.5	54.8
Vocational training	1.83	1.61	2.06	0.46	0.40	0.51	1.37	1.24	1.49	50.1	49.2	51.1	50.9	50.1	51.7
Upper secondary education	1.43	1.20	1.65	0.36	0.30	0.41	1.15	1.00	1.29	48.6	47.6	49.7	49.8	48.9	50.7
University education	1.39	1.11	1.67	0.35	0.28	0.42	1.10	0.92	1.27	48.5	47.1	49.8	49.6	48.5	50.7
Depression															
Compulsory schooling	2.73	1.91	3.55	0.46	0.32	0.59	1.70	1.34	2.06	52.7	50.3	55.2	52.4	50.3	54.5
Vocational training	2.04	1.77	2.31	0.34	0.29	0.39	1.52	1.37	1.66	50.8	49.9	51.7	50.7	49.9	51.5
Upper secondary education	1.46	1.19	1.74	0.24	0.20	0.29	1.22	1.04	1.40	49.0	47.9	50.0	49.2	48.3	50.1
University education	1.82	1.44	2.20	0.30	0.24	0.37	1.40	1.16	1.63	50.3	48.9	51.8	50.3	49.0	51.6

Anxiety															
Compulsory schooling	2.70	2.04	3.36	0.45	0.34	0.56	2.01	1.63	2.38	52.0	49.6	54.5	52.2	50.1	54.3
Vocational training	2.12	1.88	2.37	0.35	0.31	0.39	1.56	1.43	1.69	49.7	48.8	50.7	50.1	49.3	51.0
Upper secondary education	1.86	1.59	2.13	0.31	0.27	0.35	1.45	1.30	1.61	49.4	48.3	50.5	49.9	49.0	50.9
University education	2.16	1.80	2.52	0.36	0.30	0.42	1.61	1.40	1.82	50.6	49.0	52.1	50.8	49.5	52.1
Hostility															
Compulsory schooling	2.28	1.77	2.79	0.46	0.35	0.56	1.59	1.29	1.89	52.4	49.9	54.8	51.8	49.6	54.0
Vocational training	1.85	1.66	2.05	0.37	0.33	0.41	1.37	1.26	1.48	50.4	49.5	51.3	50.1	49.3	50.9
Upper secondary education	1.69	1.46	1.91	0.34	0.29	0.38	1.31	1.17	1.44	50.1	49.0	51.2	49.8	48.8	50.8
University education	1.67	1.41	1.93	0.33	0.28	0.39	1.33	1.16	1.50	49.8	48.4	51.2	49.5	48.3	50.8
Phobic anxiety															
Compulsory schooling	1.33	0.93	1.74	0.27	0.19	0.35	0.96	0.71	1.20	53.7	51.6	55.8	53.2	51.3	55.1
Vocational training	0.97	0.79	1.16	0.19	0.16	0.23	0.68	0.59	0.78	51.1	50.3	51.9	50.9	50.1	51.6
Upper secondary education	0.65	0.49	0.81	0.13	0.10	0.16	0.51	0.41	0.62	49.8	48.9	50.7	49.8	48.9	50.6
University education	0.53	0.35	0.70	0.11	0.07	0.14	0.40	0.28	0.52	48.9	47.9	50.0	48.9	47.9	49.8
Paranoid ideation															
Compulsory schooling	3.41	2.77	4.04	0.68	0.55	0.81	2.41	2.08	2.74	57.2	55.0	59.4	54.6	52.7	56.5
Vocational training	2.26	2.02	2.50	0.45	0.40	0.50	1.70	1.57	1.84	52.3	51.4	53.2	50.4	49.6	51.2
Upper secondary education	2.06	1.77	2.36	0.41	0.35	0.47	1.63	1.45	1.80	51.7	50.6	52.8	49.9	48.9	50.9
University education	1.89	1.53	2.25	0.38	0.31	0.45	1.46	1.24	1.67	50.8	49.4	52.2	49.1	47.9	50.4
Psychoticism															
Compulsory schooling	1.85	1.27	2.42	0.37	0.25	0.49	1.24	0.93	1.55	54.2	51.8	56.6	52.9	50.8	55.0
Vocational training	1.21	1.05	1.38	0.24	0.21	0.28	0.90	0.81	1.00	51.9	51.0	52.7	50.9	50.2	51.7
Upper secondary education	0.91	0.70	1.12	0.18	0.14	0.22	0.74	0.61	0.88	50.1	49.1	51.1	49.5	48.6	50.3

University education	1.03	0.77	1.30	0.21	0.15	0.26	0.79	0.63	0.95	50.8	49.5	52.1	50.0	48.9	51.1
GSI															
Compulsory schooling	25.55	20.43	30.67	0.48	0.39	0.58	17.70	15.19	20.22	53.7	50.6	56.8	52.9	50.5	55.2
Vocational training	19.54	17.78	21.30	0.37	0.34	0.40	14.75	13.84	15.67	50.1	49.0	51.3	50.3	49.4	51.2
Upper secondary education	15.63	13.82	17.44	0.29	0.26	0.33	12.62	11.59	13.65	48.5	47.2	49.7	49.1	48.1	50.1
University education	15.89	13.73	18.06	0.30	0.26	0.34	12.46	11.23	13.68	48.5	46.8	50.1	49.0	47.7	50.3
**BSI-18**															
Somatization (6 items)							**Positive Symptom Distress Index (PSDI)**								
Compulsory schooling	2.20	1.62	2.77	0.36	0.27	0.46	1.35	1.25	1.44				55.0	52.9	57.1
Vocational training	1.82	1.62	2.02	0.30	0.27	0.34	1.25	1.22	1.29				52.8	51.9	53.6
Upper secondary education	1.29	1.08	1.49	0.21	0.18	0.25	1.17	1.14	1.20				50.6	49.7	51.6
University education	1.15	0.93	1.37	0.19	0.15	0.23	1.22	1.17	1.27				49.4	48.2	50.5
GSI-18 (accepted number of missings ≤2)															
Compulsory schooling	7.63	5.93	9.32	0.42	0.33	0.52							52.4	50.1	54.6
Vocational training	5.98	5.38	6.59	0.33	0.30	0.37							50.3	49.5	51.2
Upper secondary education	4.61	4.01	5.22	0.26	0.22	0.29							49.0	48.0	50.0
University education	5.13	4.36	5.91	0.29	0.24	0.33							49.8	48.6	51.1

f) Total sample of the Swiss general population by employment

(employed, unemployed, retired)

	Sum Score			Mean Score			BSI Positive Symptom Total (PST)			T-Standardization (German norms)			T-Standardization (Swiss norms)		
	Mean	95% CI		Mean	95% CI		Mean	95% CI		Mean	95% CI		Mean	95% CI	
Somatization															
Unemployed	2.58	2.06	3.10	0.37	0.29	0.44	1.90	1.58	2.23	52.4	50.6	54.2	52.4	50.8	54.1
Employed	1.71	1.55	1.88	0.24	0.22	0.27	1.40	1.29	1.51	49.3	48.6	49.9	49.5	48.9	50.1
Retired	2.50	2.14	2.86	0.36	0.31	0.41	1.94	1.70	2.19	52.4	51.1	53.8	52.4	51.2	53.7
Obsessive-compulsive tendencies															
Unemployed	4.12	3.45	4.79	0.69	0.58	0.80	2.74	2.43	3.06	52.5	50.5	54.5	52.6	50.9	54.4
Employed	2.95	2.73	3.18	0.49	0.46	0.53	2.39	2.26	2.52	49.0	48.2	49.7	49.6	48.9	50.3
Retired	2.93	2.60	3.27	0.49	0.43	0.54	2.56	2.31	2.82	49.7	48.4	51.0	50.3	49.1	51.4
Interpersonal sensitivity															
Unemployed	2.73	2.18	3.28	0.68	0.55	0.82	1.61	1.37	1.86	53.6	51.4	55.8	53.7	51.9	55.4
Employed	1.53	1.37	1.69	0.38	0.34	0.42	1.20	1.11	1.29	48.9	48.2	49.6	49.9	49.3	50.5
Retired	1.54	1.29	1.78	0.38	0.32	0.45	1.26	1.09	1.44	49.4	48.1	50.7	50.4	49.3	51.4
Depression															
Unemployed	3.17	2.44	3.90	0.53	0.41	0.65	1.91	1.58	2.24	53.9	51.8	55.9	53.3	51.5	55.1
Employed	1.77	1.57	1.96	0.29	0.26	0.33	1.39	1.28	1.51	50.0	49.2	50.7	50.0	49.4	50.6
Retired	1.50	1.22	1.78	0.25	0.20	0.30	1.20	1.01	1.39	49.4	48.2	50.7	49.6	48.5	50.7
Anxiety															
Unemployed	3.01	2.39	3.63	0.50	0.40	0.61	1.95	1.67	2.24	52.9	50.9	54.8	52.9	51.2	54.5
Employed	2.07	1.89	2.25	0.34	0.31	0.37	1.57	1.47	1.68	50.0	49.2	50.8	50.4	49.7	51.0
Retired	1.60	1.29	1.91	0.27	0.22	0.32	1.32	1.10	1.53	47.5	46.1	49.0	48.3	47.0	49.5
Hostility															
Unemployed	2.33	1.86	2.80	0.47	0.37	0.56	1.60	1.36	1.84	52.2	50.2	54.1	51.7	49.9	53.4
Employed	1.80	1.66	1.94	0.36	0.33	0.39	1.38	1.29	1.46	50.5	49.7	51.2	50.1	49.5	50.8
Retired	1.47	1.18	1.75	0.29	0.24	0.35	1.13	0.97	1.29	48.4	47.0	49.8	48.3	47.0	49.5
Phobic anxiety															
Unemployed	1.59	1.10	2.09	0.32	0.22	0.42	0.93	0.71	1.16	53.4	51.5	55.3	52.8	51.1	54.5
Employed	0.71	0.59	0.82	0.14	0.12	0.16	0.54	0.47	0.60	49.9	49.4	50.5	49.8	49.3	50.4
Retired	0.81	0.64	0.99	0.16	0.13	0.20	0.68	0.55	0.82	51.0	49.9	52.1	50.8	49.8	51.9

Paranoid ideation															
Unemployed	3.00	2.45	3.56	0.60	0.49	0.71	2.03	1.77	2.29	54.9	53.1	56.7	52.8	51.2	54.4
Employed	2.16	1.97	2.35	0.43	0.39	0.47	1.66	1.55	1.77	52.0	51.2	52.7	50.1	49.4	50.7
Retired	1.86	1.59	2.14	0.37	0.32	0.43	1.52	1.33	1.71	51.3	50.1	52.6	49.6	48.5	50.6
Psychoticism															
Unemployed	1.89	1.42	2.36	0.38	0.28	0.47	1.17	0.94	1.40	54.3	52.4	56.3	53.0	51.3	54.7
Employed	1.09	0.95	1.22	0.22	0.19	0.24	0.84	0.76	0.92	51.1	50.4	51.7	50.2	49.7	50.8
Retired	0.90	0.73	1.07	0.18	0.15	0.21	0.77	0.64	0.90	50.6	49.6	51.7	50.0	49.0	50.9
GSI															
Unemployed	26.58	22.02	31.14	0.50	0.42	0.59	17.23	15.24	19.22	54.0	51.7	56.4	53.4	51.5	55.2
Employed	17.23	15.97	18.50	0.33	0.30	0.35	13.50	12.81	14.19	48.9	48.0	49.8	49.3	48.6	50.0
Retired	16.85	14.93	18.77	0.32	0.28	0.35	13.72	12.40	15.05	49.6	48.0	51.1	49.9	48.6	51.1
**BSI-18**															
Somatization (6 items)							**Positive Symptom Distress Index (PSDI)**								
Unemployed	2.05	1.62	2.48	0.34	0.27	0.41	1.38	1.30	1.46				53.9	52.2	55.6
Employed	1.43	1.28	1.57	0.24	0.21	0.26	1.22	1.20	1.24				50.9	50.3	51.6
Retired	2.07	1.76	2.37	0.34	0.29	0.40	1.18	1.15	1.22				53.9	52.6	55.2
GSI-18 (accepted number of missings ≤2)															
Unemployed	8.23	6.66	9.80	0.46	0.37	0.54							52.9	51.1	54.7
Employed	5.26	4.83	5.70	0.29	0.27	0.32							49.6	48.9	50.2
Retired	5.17	4.46	5.87	0.29	0.25	0.33							49.9	48.7	51.1

g) *Total sample of the Swiss general population by migration background*

	Sum Score			Mean Score			BSI Positive Symptom Total (PST)			T-Standardization (German norms)			T-Standardization (Swiss norms)		
	Mean	95% CI		Mean	95% CI		Mean	95% CI		Mean	95% CI		Mean	95% CI	
Somatization															
No migration background	1.97	1.81	2.13	0.28	0.26	0.30	1.56	1.46	1.67	50.4	49.8	51.0	50.5	50.0	51.1
Migration background	1.96	1.63	2.29	0.28	0.23	0.33	1.57	1.34	1.79	49.9	48.6	51.2	50.1	48.9	51.2
Obsessive-compulsive tendencies															
No migration background	3.03	2.82	3.23	0.50	0.47	0.54	2.38	2.26	2.49	49.3	48.6	49.9	49.8	49.2	50.4
Migration background	3.34	2.93	3.76	0.56	0.49	0.63	2.66	2.42	2.90	50.3	49.0	51.7	50.8	49.6	52.0
Interpersonal sensitivity															
No migration background	1.68	1.53	1.84	0.42	0.38	0.46	1.24	1.16	1.33	49.5	48.8	50.2	50.4	49.9	51.0
Migration background	1.77	1.48	2.05	0.44	0.37	0.51	1.37	1.19	1.55	50.1	48.7	51.4	50.8	49.7	51.9
Depression															
No migration background	1.87	1.68	2.07	0.31	0.28	0.34	1.40	1.29	1.51	50.2	49.6	50.9	50.2	49.7	50.8
Migration background	2.10	1.72	2.47	0.35	0.29	0.41	1.56	1.34	1.77	51.2	49.8	52.5	51.0	49.9	52.2
Anxiety															
No migration background	2.02	1.85	2.19	0.34	0.31	0.37	1.52	1.43	1.62	49.6	48.9	50.3	50.1	49.5	50.7
Migration background	2.44	2.08	2.79	0.41	0.35	0.47	1.78	1.58	1.98	51.3	49.9	52.8	51.4	50.2	52.6
Hostility															
No migration background	1.86	1.72	2.00	0.37	0.34	0.40	1.40	1.32	1.47	50.7	50.0	51.3	50.3	49.7	50.9
Migration background	1.76	1.50	2.03	0.35	0.30	0.41	1.32	1.16	1.48	49.9	48.5	51.2	49.6	48.4	50.8
Phobic anxiety															
No migration background	0.88	0.75	1.00	0.18	0.15	0.20	0.63	0.56	0.70	50.7	50.2	51.3	50.6	50.1	51.1
Migration background	0.78	0.58	0.98	0.16	0.12	0.20	0.60	0.46	0.73	50.3	49.2	51.4	50.1	49.1	51.1
Paranoid ideation															
No migration background	2.13	1.96	2.30	0.43	0.39	0.46	1.62	1.52	1.72	51.9	51.2	52.5	50.0	49.4	50.6

Migration background	2.53	2.18	2.89	0.51	0.44	0.58	1.92	1.72	2.13	53.4	52.1	54.7	51.5	50.3	52.6
Psychoticism															
No migration background	1.13	1.00	1.25	0.23	0.20	0.25	0.84	0.77	0.91	51.3	50.7	51.9	50.5	50.0	51.0
Migration background	1.31	1.04	1.58	0.26	0.21	0.32	0.98	0.83	1.14	52.1	50.8	53.3	51.1	50.0	52.2
GSI															
No migration background	18.18	16.93	19.43	0.34	0.32	0.37	13.81	13.16	14.46	49.6	48.8	50.4	49.9	49.3	50.5
Migration background	19.50	17.10	21.90	0.37	0.32	0.41	14.94	13.62	16.25	50.2	48.5	51.9	50.4	49.1	51.7
**BSI-18**															
Somatization (6 items)							**Positive Symptom Distress Index (PSDI)**								
No migration background	1.63	1.49	1.76	0.27	0.25	0.29	1.23	1.20	1.25				52.0	51.4	52.6
Migration background	1.62	1.34	1.91	0.27	0.22	0.32	1.26	1.22	1.30				51.5	50.3	52.7
GSI-18 (accepted number of missings ≤2)															
No migration background	5.52	5.11	5.94	0.31	0.28	0.33							50.0	49.4	50.6
Migration background	6.16	5.29	7.03	0.34	0.29	0.39							50.6	49.3	51.8
