# Supplementary material for: The Brief Symptom Inventory in the Swiss general population: Presentation of norm scores and predictors of psychological distress
Source: PLoS One. 2024 Jul 3;19(7):e0305192. doi: 10.1371/journal.pone.0305192 (PMC11221686; doi:10.1371/journal.pone.0305192)
Supplement: S4 Appendix — (PDF) [file pone.0305192.s006.pdf]

Psychometric properties of the Brief Symptom Inventory in the Swiss general population: Presentation of norm scores and predictors of psychological distress

Gisela Michel [1*], Julia Baenziger [1], Jeannette Brodbeck [2], Luzius Mader [1,3,4], Claudia Kuehni [3,5], Katharina Roser [1]

[1] Faculty of Health Sciences and Medicine, University of Lucerne, Alpenquai 4, 6005 Lucerne, Switzerland; E-mail: gisela.michel@unilu.ch, julia.baenziger@outlook.com, katharina.roser@unilu.ch

[2] Institute of Psychology, University of Bern, Fabrikstrasse 8, 3012 Bern, Switzerland. E-mail: jeannette.brodbeck@unibe.ch

[3] Institute for Social and Preventive Medicine, University of Bern, Mittelstrasse 43, 3012 Bern, Switzerland. E-mail: claudia.kuehni@ispm.unibe.ch

[4] Cancer Registry Bern-Solothurn, University of Bern, Murtenstrasse 31, 3008 Bern, Switzerland. E-mail: luzius.mader@unibe.ch

[5] Pediatric Hematology and Oncology, University Children's Hospital Bern, University of Bern, Freiburgstrasse 15, 3010 Bern, Bern, Switzerland.

*Corresponding author: Gisela Michel, Faculty of Health Sciences and Medicine, University of Lucerne, Alpenquai 4, 6005 Lucerne, Switzerland, E-mail: gisela.michel@unilu.ch

Appendix D: Supplemental Tables

S11 Table: Characteristics associated with psychological distress in the Swiss general population (univariable regression analyses; statistically significant p-values highlighted in red)........ 2

S12 Table: Characteristics associated with psychological distress in the Swiss general population (multivariable regression using the weighted dataset; including characteristics which were significantly associated at p<0.05 in the univariable regression; statistically significant p-values highlighted in red) 5

S13 Table: Proportions of participants being cases with psychological distress (GSI T≥63 or 2 scales T≥63) and differences between different groups (from univariable logistic regression with weighted dataset) 8

S11 Table: Characteristics associated with psychological distress in the Swiss general population (univariable regression analyses; statistically significant p-values highlighted in red)

	Somatization				Obsessive-compulsive tendencies				Interpersonal Sensitivity				Depression			
	Coef	95% CI		p	Coef	95% CI		p	Coef	95% CI		p	Coef	95% CI		p
**Sex**	F(1,1236)=31.26, p<0.001				F(1,1234)=3.1, p=0.0785				F(1,1237)=24.97, p<0.001				F(1,1236)=2.56, p=0.110			
Male	1.5	1.3	1.7		2.9	2.7	3.2		1.3	1.2	1.5		1.8	1.5	2.1	
Female	0.8	0.5	1.1	<0.001	0.3	0.0	0.7	0.079	0.7	0.4	1.0	<0.001	0.3	-0.1	0.6	0.110
**Age group**	F(5,1232)=5.460, p<0.001				F(5,1230)=2.170, p=0.055				F(5,1233)=2.950, p=0.012				F(5,1232)=3.480, p=0.004			
18-25 years	2.4	1.8	3.0		4.4	3.6	5.3		2.7	2.1	3.4		2.9	2.2	3.7	
26-35 years	-1.0	-1.7	-0.3	0.006	-1.3	-2.3	-0.3	0.010	-0.9	-1.7	-0.1	0.019	-0.5	-1.5	0.4	0.276
36-45 years	-0.8	-1.5	-0.1	0.026	-1.4	-2.3	-0.4	0.004	-1.0	-1.7	-0.3	0.007	-1.1	-1.9	-0.2	0.013
46-55 years	-0.3	-1.0	0.3	0.323	-1.4	-2.3	-0.4	0.004	-1.2	-2.0	-0.5	0.001	-1.2	-2.0	-0.4	0.005
56-65 years	-0.1	-0.9	0.6	0.736	-1.5	-2.4	-0.6	0.002	-1.2	-1.9	-0.5	0.001	-1.4	-2.2	-0.6	0.001
66-75 years	0.1	-0.7	0.8	0.866	-1.5	-2.4	-0.5	0.002	-1.2	-1.9	-0.5	0.001	-1.4	-2.2	-0.5	0.002
**Language Region**	F(2,1235)=5.170, p=0.006				F(2,1233)=1.570, p=0.208				F(2,1236)=2.550, p=0.079				F(2,1235)=4.900, p=0.008			
German / Romansh	1.9	1.7	2.1		3.2	2.9	3.4		1.7	1.6	1.9		2.0	1.8	2.2	
French	-0.1	-0.5	0.2	0.539	-0.3	-0.8	0.2	0.239	-0.3	-0.6	0.1	0.116	-0.4	-0.8	0.0	0.038
Italian	1.5	0.5	2.4	0.002	0.6	-0.4	1.6	0.228	0.6	-0.2	1.3	0.145	1.1	0.1	2.1	0.034
**Education**	F(3,1162)=14.250, p<0.001				F(3,1160)=6.890, p<0.001				F(3,1163)=5.140, p=0.002				F(3,1162)=4.720, p=0.003			
Compulsory schooling	2.9	2.2	3.6		4.2	3.3	5.1		2.4	1.8	3.1		2.8	1.9	3.6	
Vocational training	-0.7	-1.4	0.1	0.068	-0.9	-1.8	0.0	0.056	-0.6	-1.3	0.1	0.087	-0.7	-1.6	0.1	0.100
Upper secondary education	-1.3	-2.1	-0.6	<0.001	-1.7	-2.6	-0.7	<0.001	-1.0	-1.7	-0.3	0.004	-1.3	-2.2	-0.4	0.003
University education	-1.6	-2.3	-0.9	<0.001	-1.3	-2.3	-0.4	0.007	-1.0	-1.7	-0.3	0.004	-0.9	-1.8	0.0	0.041
**Employment**	F(2,1203)=10.740, p<0.001				F(2,1201)=5.390, p=0.005				F(2,1204)=8.370, p<0.001				F(2,1203)=8.660, p<0.001			
Unemployed	2.6	2.1	3.1		4.1	3.4	4.8		2.7	2.2	3.3		3.2	2.4	3.9	
Employed	-0.9	-1.4	-0.3	0.002	-1.2	-1.9	-0.4	0.001	-1.2	-1.7	-0.6	<0.001	-1.4	-2.1	-0.6	<0.001
Retired	-0.1	-0.7	0.6	0.809	-1.2	-1.9	-0.4	0.002	-1.2	-1.8	-0.6	<0.001	-1.6	-2.4	-0.9	<0.001
**Migration background**	F(1,1236)=0.000, p=0.957				F(1,1234)=1.670, p=0.197				F(1,1237)=0.200, p=0.654				F(1,1236)=1.020, p=0.313			
No migration background	2.0	1.8	2.1		3.0	2.8	3.2		1.7	1.5	1.8		1.9	1.7	2.1	
Migration background	0.0	-0.4	0.4	0.957	0.3	-0.2	0.8	0.197	0.1	-0.3	0.4	0.654	0.2	-0.2	0.6	0.313

S11 Table contd.

	Anxiety				Hostility				Phobic anxiety				Paranoid ideation			
	Coef	95% CI		p	Coef	95% CI		p	Coef	95% CI		p	Coef	95% CI		p
**Sex**	F(1,1236)=11.2, p<0.001				F(1,1236)=4.27, p=0.039				F(1,1237)=1.45, p=0.2285				F(1,1236)=0.91, p=0.3408			
Male	1.9	1.6	2.1		1.7	1.5	1.9		0.8	0.6	1.0		2.2	1.9	2.4	
Female	0.5	0.2	0.9	0.001	0.3	0.0	0.5	0.039	0.1	-0.1	0.3	0.228	0.2	-0.2	0.5	0.341
**Age group**	F(5,1232)=4.760, p<0.001				F(5,1232)=3.690, p=0.003				F(5,1233)=2.230, p=0.049				F(5,1232)=2.820, p=0.015			
18-25 years	2.9	2.3	3.6		2.5	2.0	3.0		1.5	1.0	2.0		2.9	2.3	3.6	
26-35 years	-0.5	-1.2	0.3	0.227	-0.6	-1.2	0.0	0.044	-0.7	-1.3	-0.1	0.031	-0.7	-1.5	0.0	0.060
36-45 years	-0.7	-1.5	0.0	0.049	-0.5	-1.1	0.1	0.097	-0.8	-1.3	-0.2	0.004	-0.8	-1.5	0.0	0.053
46-55 years	-0.9	-1.7	-0.2	0.010	-0.9	-1.5	-0.3	0.002	-0.8	-1.4	-0.3	0.004	-0.7	-1.5	0.0	0.050
56-65 years	-0.9	-1.6	-0.2	0.016	-0.9	-1.5	-0.4	0.001	-0.5	-1.1	0.0	0.066	-0.5	-1.2	0.2	0.188
66-75 years	-1.4	-2.1	-0.7	<0.001	-1.0	-1.6	-0.4	0.001	-0.7	-1.2	-0.1	0.015	-1.1	-1.8	-0.4	0.002
**Language Region**	F(2,1235)=9710, p<0.001				F(2,1235)=3.980, p=0.019				F(2,1236)=0.110, p=0.900				F(2,1235)=3.810, p=0.023			
German / Romansh	1.9	1.8	2.1		1.8	1.6	1.9		0.9	0.7	1.0		2.1	2.0	2.3	
French	0.5	0.1	1.0	0.014	0.0	-0.3	0.4	0.797	-0.1	-0.3	0.2	0.646	0.3	-0.1	0.8	0.135
Italian	1.7	0.8	2.6	<0.001	1.1	0.3	1.8	0.005	0.0	-0.6	0.6	0.940	1.1	0.2	1.9	0.015
**Education**	F(3,1162)=2.320, p=0.074				F(3,1162)=1.900, p=0.128				F(3,1163)=7.440, p<0.001				F(3,1162)=6.150, p<0.001			
Compulsory schooling	2.8	2.1	3.4		2.3	1.8	2.8		1.3	0.9	1.7		3.4	2.8	4.1	
Vocational training	-0.6	-1.3	0.1	0.083	-0.4	-1.0	0.1	0.128	-0.4	-0.8	0.1	0.104	-1.2	-1.8	-0.5	0.001
Upper secondary education	-0.9	-1.6	-0.2	0.014	-0.6	-1.2	0.0	0.036	-0.7	-1.1	-0.3	0.002	-1.4	-2.1	-0.7	<0.001
University education	-0.6	-1.4	0.2	0.124	-0.6	-1.2	0.0	0.036	-0.8	-1.3	-0.4	<0.001	-1.5	-2.3	-0.8	<0.001
**Employment**	F(2,1204)=8.280, p<0.001				F(2,1203)=4.950, p=0.007				F(2,1204)=6.070, p=0.002				F(2,1203)=6.700, p=0.001			
Unemployed	3.0	2.4	3.6		2.3	1.9	2.8		1.6	1.1	2.1		3.0	2.4	3.5	
Employed	-0.9	-1.6	-0.3	0.005	-0.5	-1.0	0.0	0.038	-0.9	-1.4	-0.4	0.001	-0.8	-1.4	-0.2	0.006
Retired	-1.4	-2.1	-0.7	<0.001	-0.9	-1.4	-0.3	0.002	-0.8	-1.3	-0.2	0.005	-1.1	-1.7	-0.5	<0.001
**Migration background**	F(1,1236)=4.050, p=0.044				F(1,1236)=0.350, p=0.557				F(1,1237)=0.770, p=0.379				F(1,1236)=3.750, p=0.053			
No migration background	2.0	1.9	2.2		1.9	1.7	2.0		0.9	0.8	1.0		2.1	2.0	2.3	
Migration background	0.4	0.0	0.8	0.044	-0.1	-0.4	0.2	0.557	-0.1	-0.3	0.1	0.379	0.4	0.0	0.8	0.053

S11 Table contd.

	Psychoticism				GSI				Somatization (6 items)				GSI-18			
	Coef	95% CI		p	Coef	95% CI		p	Coef	95% CI		p	Coef	95% CI		p
**Sex**	F(1,1236)=1.41, p=0.236				F(1,1231)=10.84, p=0.001				F(1,1236)=10.81, p<0.001				F(1,1234)=9.89, p=0.002			
Male	1.1	0.9	1.3		16.6	14.9	18.3		1.4	1.2	1.6		5.1	4.5	5.6	
Female	0.1	-0.1	0.4	0.236	3.8	1.5	6.1	0.001	0.4	0.2	0.7	0.001	1.3	0.5	2.0	0.002
**Age group**	F(5,1232)=3.250, p=0.006				F(5,1227)=2.560, p=0.026				F(5,1232)=3.780, p=0.002				F(5,1230)=2.090, p=0.064			
18-25 years	2.0	1.5	2.5		26.3	21.4	31.3		2.0	1.4	2.5		7.9	6.3	9.5	
26-35 years	-0.8	-1.4	-0.2	0.009	-7.3	-13.0	-1.6	0.012	-0.7	-1.3	-0.1	0.016	-1.7	-3.6	0.2	0.077
36-45 years	-0.8	-1.4	-0.3	0.004	-8.4	-13.9	-2.9	0.003	-0.6	-1.2	0.0	0.052	-2.4	-4.2	-0.6	0.010
46-55 years	-1.0	-1.5	-0.4	0.001	-9.0	-14.5	-3.5	0.001	-0.4	-0.9	0.2	0.227	-2.5	-4.3	-0.7	0.006
56-65 years	-1.0	-1.6	-0.4	<0.001	-8.5	-14.0	-2.9	0.003	-0.2	-0.8	0.4	0.533	-2.5	-4.3	-0.6	0.008
66-75 years	-1.1	-1.6	-0.5	<0.001	-9.5	-15.0	-4.1	0.001	0.1	-0.6	0.7	0.835	-2.7	-4.5	-0.9	0.003
**Language Region**	F(2,1235)=1.140, p=0.321				F(2,1230)=3.15, p=0.043				F(2,1235)=5.410, p=0.005				F(2,1233)=5.570, p=0.004			
German / Romansh	1.1	1.0	1.3		18.2	17.0	19.5		1.6	1.4	1.7		5.5	5.0	5.9	
French	0.0	-0.3	0.3	0.940	-0.6	-3.4	2.2	0.670	-0.1	-0.4	0.3	0.738	0.1	-0.9	1.1	0.888
Italian	0.6	-0.2	1.3	0.132	8.7	1.7	15.8	0.015	1.3	0.5	2.1	0.001	4.2	1.7	6.6	0.001
**Education**	F(3,1162)=3.960, p=0.008				F(3,1157)=6.960, p<0.001				F(3,1162)=9.910, p<0.001				F(3,1160)=5.920, p=0.001			
Compulsory schooling	1.9	1.3	2.4		25.6	20.4	30.7		2.2	1.6	2.8		7.7	6.0	9.4	
Vocational training	-0.6	-1.2	0.0	0.034	-6.0	-11.4	-0.6	0.030	-0.4	-1.0	0.2	0.195	-1.8	-3.6	0.0	0.056
Upper secondary education	-0.9	-1.6	-0.3	0.002	-9.9	-15.4	-4.5	<0.001	-0.9	-1.5	-0.3	0.003	-3.1	-4.9	-1.3	0.001
University education	-0.8	-1.5	-0.2	0.011	-9.7	-15.2	-4.1	0.001	-1.1	-1.7	-0.5	0.001	-2.6	-4.5	-0.7	0.006
**Employment**	F(2,1203)=7.670, p=0.001				F(2,1199)=7.910, p<0.001				F(2,1203)=9.060, p<0.001				F(2,1202)=6.490, p=0.002			
Unemployed	1.9	1.4	2.3		26.6	22.0	31.1		2.0	1.6	2.5		8.2	6.6	9.8	
Employed	-0.8	-1.3	-0.3	0.001	-9.3	-14.1	-4.6	<0.001	-0.6	-1.1	-0.2	0.008	-2.9	-4.5	-1.3	<0.001
Retired	-1.0	-1.5	-0.5	<0.001	-9.7	-14.7	-4.8	<0.001	0.0	-0.5	0.6	0.933	-3.0	-4.7	-1.3	0.001
**Migration background**	F(1,1236)=1.490, p=0.223				F(1,1231)=0.920, p=0.339				F(1,1236)=0.000, p=0.995				F(1,1234)=1.610, p=0.205			
No migration background	1.1	1.0	1.2		18.2	16.9	19.4		1.6	1.5	1.8		5.5	5.1	6.0	
Migration background	0.2	-0.1	0.5	0.223	1.3	-1.4	4.0	0.339	0.0	-0.3	0.3	0.995	0.6	-0.3	1.6	0.205

F statistic tests provides an overall test for the hypothesis that all coefficients excluding the constant are zero.

S12 Table: Characteristics associated with psychological distress in the Swiss general population (multivariable regression using the weighted dataset; including characteristics which were significantly associated at p<0.05 in the univariable regression; statistically significant p-values highlighted in red)

	Somatization				Obsessive-compulsive tendencies				Interpersonal Sensitivity				Depression			
	F(13,1146)=7.45				F(5,1152)=5.580				F(11,1149)=6.100				F(12,1147)=4.04			
	p<0.001				p<0.001				p<0.001				p<0.001			
	**Coef**	**95% CI**		**p**	**Coef**	**95% CI**		**p**	**Coef**	**95% CI**		**p**	**Coef**	**95% CI**		**p**
**Sex**																
Female	0.8	0.5	1.0	<0.001					0.6	0.3	0.9	<0.001				
**Age group**																
26-35 years	-0.4	-1.1	0.4	0.367					-0.4	-1.2	0.5	0.420	0.1	-0.9	1.2	0.800
36-45 years	-0.1	-0.9	0.7	0.781					-0.3	-1.1	0.5	0.421	-0.3	-1.2	0.6	0.551
46-55 years	0.2	-0.6	1.0	0.565					-0.6	-1.4	0.2	0.147	-0.5	-1.4	0.5	0.322
56-65 years	0.2	-0.6	0.9	0.677					-0.9	-1.6	-0.1	0.026	-1.0	-1.8	-0.1	0.027
66-75 years	-0.3	-1.3	0.7	0.594					-1.1	-2.0	-0.2	0.016	-1.0	-2.0	0.0	0.060
**Language region**																
French	-0.1	-0.5	0.2	0.470									-0.5	-0.9	-0.1	0.016
Italian	0.8	0.2	1.5	0.015									0.6	-0.5	1.6	0.278
**Education**																
Vocational training	-0.4	-1.2	0.3	0.236	-0.8	-1.7	0.1	0.098	-0.3	-1.0	0.4	0.382	-0.6	-1.4	0.3	0.194
Upper secondary education	-0.9	-1.6	-0.2	0.018	-1.5	-2.4	-0.5	0.002	-0.6	-1.2	0.1	0.114	-1.0	-1.9	-0.2	0.019
University education	-1.1	-1.8	-0.3	0.005	-1.2	-2.2	-0.2	0.022	-0.6	-1.3	0.1	0.115	-0.7	-1.6	0.2	0.125
**Employment**																
Employed	-0.5	-1.2	0.1	0.113	-1.0	-1.7	-0.2	0.011	-0.9	-1.5	-0.2	0.009	-1.2	-2.1	-0.4	0.006
Retired	0.2	-0.8	1.2	0.693	-1.2	-1.9	-0.4	0.003	-0.4	-1.2	0.4	0.301	-1.0	-1.9	0.0	0.047
**Migration background**																
Migration background																
**Intercept**	2.6	1.7	3.4		4.9	3.9	6.0		3.0	2.1	3.9		4.1	3.0	5.2	

S12 Table contd.

	**Anxiety**				**Hostility**				**Phobic anxiety**				**Paranoid ideation**			
	F(11,1195)=6.540				F(10,1195)=3.740				F(10,1150)=3.090				F(12,1147)=3.360			
	p<0.001				p<0.001				p=0.001				p<0.001			
	**Coef**	**95% CI**		**p**	**Coef**	**95% CI**		**p**	**Coef**	**95% CI**		**p**	**Coef**	**95% CI**		**p**
**Sex**																
Female	0.5	0.2	0.8	0.004	0.2	0.0	0.5	0.109								
**Age group**																
26-35 years	-0.3	-1.2	0.5	0.439	-0.6	-1.3	0.1	0.080	-0.2	-0.9	0.5	0.546	-0.3	-1.1	0.5	0.497
36-45 years	-0.5	-1.3	0.3	0.210	-0.4	-1.0	0.2	0.227	-0.3	-0.9	0.3	0.352	-0.2	-1.0	0.7	0.674
46-55 years	-0.7	-1.5	0.1	0.097	-0.8	-1.4	-0.1	0.020	-0.3	-1.0	0.3	0.297	-0.2	-1.1	0.6	0.592
56-65 years	-0.8	-1.5	0.0	0.052	-0.9	-1.5	-0.2	0.007	-0.3	-0.9	0.3	0.316	-0.2	-1.0	0.6	0.618
66-75 years	-1.7	-2.7	-0.7	0.001	-0.8	-1.5	-0.1	0.020	-0.5	-1.2	0.2	0.136	-0.8	-1.7	0.1	0.096
**Language region**																
French	0.5	0.1	0.9	0.018	0.1	-0.3	0.4	0.765					0.3	-0.1	0.8	0.145
Italian	1.7	0.7	2.7	0.001	1.2	0.4	1.9	0.005					0.8	-0.1	1.7	0.068
**Education**																
Vocational training									-0.2	-0.7	0.2	0.303	-1.0	-1.7	-0.3	0.004
Upper secondary education									-0.5	-0.9	0.0	0.039	-1.1	-1.9	-0.4	0.002
University education									-0.6	-1.0	-0.1	0.010	-1.3	-2.1	-0.6	<0.001
**Employment**																
Employed	-0.6	-1.3	0.1	0.113	-0.3	-0.9	0.3	0.306	-0.7	-1.3	-0.1	0.018	-0.6	-1.2	0.1	0.090
Retired	-0.1	-1.1	0.9	0.850	-0.4	-1.1	0.2	0.170	-0.4	-1.1	0.3	0.227	-0.6	-1.4	0.3	0.188
**Migration background**																
Migration background	0.2	-0.2	0.6	0.389												
**Intercept**	2.8	2.0	3.5		2.5	1.9	3.1		2.0	1.4	2.7		3.9	3.1	4.8	

S12 Table contd.

	Psychoticism				GSI				Somatization (6 items)				GSI-18			
	F(10,1149)=3.540				F(11,1144)=4.440				F(13,1146)=4.620				F(8,1150)=5.550			
	p<0.001				p<0.001				p<0.001				p<0.001			
	**Coef**	**95% CI**		**p**	**Coef**	**95% CI**		**p**	**Coef**	**95% CI**		**p**	**Coef**	**95% CI**		**p**
**Sex**																
Female					3.0	0.7	5.3	0.010	0.4	0.1	0.6	0.002	1.1	0.4	1.9	0.004
**Age group**																
26-35 years	-0.4	-1.0	0.3	0.253	-2.8	-9.4	3.8	0.407	-0.3	-1.0	0.3	0.341				
36-45 years	-0.4	-1.0	0.3	0.237	-3.0	-9.2	3.3	0.350	-0.1	-0.8	0.6	0.723				
46-55 years	-0.5	-1.2	0.1	0.111	-4.0	-10.5	2.5	0.229	0.0	-0.7	0.7	0.946				
56-65 years	-0.8	-1.4	-0.2	0.007	-5.8	-11.8	0.2	0.058	-0.1	-0.7	0.6	0.878				
66-75 years	-0.9	-1.6	-0.2	0.008	-8.3	-15.2	-1.5	0.016	-0.4	-1.3	0.5	0.365				
**Language region**																
French					-0.7	-3.6	2.1	0.618	-0.1	-0.4	0.3	0.714	0.0	-1.0	1.0	0.988
Italian					5.4	-1.1	11.9	0.106	0.8	0.2	1.4	0.011	2.8	0.6	5.0	0.011
**Education**																
Vocational training	-0.5	-1.1	0.1	0.094	-4.0	-9.4	1.4	0.145	-0.2	-0.8	0.4	0.473	-1.2	-3.0	0.7	0.209
Upper secondary education	-0.7	-1.3	-0.1	0.018	-6.7	-12.2	-1.2	0.017	-0.6	-1.2	0.0	0.051	-2.2	-4.1	-0.3	0.021
University education	-0.6	-1.2	0.0	0.050	-6.4	-12.0	-0.7	0.026	-0.7	-1.3	-0.1	0.027	-1.6	-3.5	0.3	0.100
**Employment**																
Employed	-0.6	-1.1	0.0	0.040	-6.9	-12.6	-1.2	0.018	-0.4	-0.9	0.2	0.167	-2.4	-4.2	-0.7	0.006
Retired	-0.5	-1.1	0.2	0.148	-4.1	-10.6	2.4	0.216	0.3	-0.5	1.1	0.470	-2.9	-4.7	-1.2	0.001
**Migration background**																
Migration background																
**Intercept**	2.7	1.9	3.5		31.3	24.3	38.2		2.1	1.4	2.9		8.5	6.4	10.6	

F statistic tests provides an overall test for the hypothesis that all coefficients excluding the constant are zero.

S13 Table: Proportions of participants being cases with psychological distress (GSI T≥63 or 2 scales T≥63) and differences between different groups (from univariable logistic regression with weighted dataset)

	No distress				Cases with distress								
	%	95% CI			%	95% CI			OR	95% CI			p
Total	81.9	79.5	-	84.1	18.1	16.0	-	20.5					
**Sex**													<0.001
Male	86.1	82.6	-	89.0	13.9	11.0	-	17.4					
Female	77.9	74.5	-	80.9	22.1	19.1	-	25.5	1.76	1.27	-	2.44	
**Age group**													0.019
18-25 years	69.5	59.3	-	78.1	30.5	21.9	-	40.7					
26-35 years	80.5	73.3	-	86.1	19.5	13.9	-	26.7	0.55	0.30	-	1.01	
36-45 years	81.5	75.5	-	86.3	18.5	13.7	-	24.5	0.52	0.29	-	0.92	
46-55 years	86.2	81.8	-	89.7	13.8	10.3	-	18.2	0.36	0.21	-	0.64	
56-65 years	83.5	78.0	-	87.9	16.5	12.1	-	22.0	0.45	0.25	-	0.80	
66-75 years	83.3	77.3	-	87.9	16.7	12.1	-	22.7	0.46	0.25	-	0.82	
**Language region**													0.253
German	82.9	80.2	-	85.3	17.1	14.7	-	19.8					
French	80.3	74.6	-	85.0	19.7	15.0	-	25.4	1.19	0.82	-	1.73	
Italian	75.1	62.5	-	84.5	24.9	15.5	-	37.5	1.61	0.87	-	2.98	
**Education**													<0.001
Compulsory schooling	69.0	58.6	-	77.8	31.0	22.2	-	41.4					
Vocational training	80.2	76.5	-	83.5	19.8	16.5	-	23.5	0.55	0.33	-	0.91	
Upper secondary education	87.0	82.6	-	90.4	13.0	9.6	-	17.4	0.33	0.19	-	0.59	
University education	86.9	81.1	-	91.1	13.1	8.9	-	18.9	0.34	0.18	-	0.63	
**Employment**													<0.001
Unemployed	68.6	60.2	-	76.0	31.4	24.0	-	39.8					
Employed	84.8	82.0	-	87.2	15.2	12.8	-	18.0	0.39	0.26	-	0.60	
Retired	82.0	76.0	-	86.8	18.0	13.2	-	24.0	0.48	0.28	-	0.81	
**Migration background**													0.154
No migration background	83.1	80.5	-	85.3	16.9	14.7	-	19.5					
Migration background	79.1	73.5	-	83.8	20.9	16.2	-	26.5	1.29	0.91	-	1.85	

OR odds ratio, CI confidence interval
