# Supplementary material for: The Brief Symptom Inventory in the Swiss general population: Presentation of norm scores and predictors of psychological distress
Source: PLoS One. 2024 Jul 3;19(7):e0305192. doi: 10.1371/journal.pone.0305192 (PMC11221686; doi:10.1371/journal.pone.0305192)
Supplement: S5 Appendix — (PDF) [file pone.0305192.s007.pdf]

Psychometric properties of the Brief Symptom Inventory in the Swiss general population: Presentation of norm scores and predictors of psychological distress

Gisela Michel [1*], Julia Baenziger [1], Jeannette Brodbeck [2], Luzius Mader [1,3,4], Claudia Kuehni [3,5], Katharina Roser [1]

[1] Faculty of Health Sciences and Medicine, University of Lucerne, Alpenquai 4, 6005 Lucerne, Switzerland; E-mail: gisela.michel@unilu.ch, julia.baenziger@outlook.com, katharina.roser@unilu.ch
[2] Institute of Psychology, University of Bern, Fabrikstrasse 8, 3012 Bern, Switzerland. E-mail: jeannette.brodbeck@unibe.ch
[3] Institute for Social and Preventive Medicine, University of Bern, Mittelstrasse 43, 3012 Bern, Switzerland. E-mail: claudia.kuehni@ispm.unibe.ch
[4] Cancer Registry Bern-Solothurn, University of Bern, Murtenstrasse 31, 3008 Bern, Switzerland. E-mail: luzius.mader@unibe.ch
[5] Pediatric Hematology and Oncology, University Children's Hospital Bern, University of Bern, Freiburgstrasse 15, 3010 Bern, Bern, Switzerland.

*Corresponding author: Gisela Michel, Faculty of Health Sciences and Medicine, University of Lucerne, Alpenquai 4, 6005 Lucerne, Switzerland, E-mail: Gisela.michel@unilu.ch

Appendix E: Supplemental Tables

S14 Table: Comparison of the mean scores of the Swiss general population (weighted sample) with three different German norm populations using independent t-tests 2

S15 Table: Comparison of different T-standardizations of the Swiss general population data using paired t-tests 5

References:

Franke, G.H., *BSI Brief Symptom Inventory - deutsche Fassung Brief Symptom Inventory (BSI; Derogatis, L.R. & Melisaratos, N., 1983) - German version/author.* 2000.

Franke, G.F., *BSCL - Brief-Symptom-Checklist*. 2017, Göttingen: Hogrefe.

Kliem, S. and E. Braehler, *BSI - Brief Symptom Inventory (Kurzform des Symptom-Checklist-90-R) von L. R. Derogatis, deutsche Fassung - Manual (1. Auflage)*. 2017, Frankfurt am Main: Pearson.

S14 Table: Comparison of the mean scores of the Swiss general population (weighted sample) with three different German norm populations using independent t-tests

	Swiss general population			German population, Franke 2000					German population, Kliem & Braehler, 2017					German population, Franke, 2017				
	N	mean	SD	mean	SD	t	df	p	mean	SD	t	df	p	mean	SD	t	df	p
**Total population**																		
Somatization	1237	0.28	0.38	0.27	0.32	0.65	1835	0.514	0.37	0.45	-4.97	2201	**<0.001**	0.38	0.49	-6.02	3260	**<0.001**
Obsessive-compulsive tendencies	1235	0.52	0.57	0.52	0.44	0.04	1833	0.967	0.57	0.59	-1.97	2199	**0.050**	0.58	0.62	-2.71	3258	**0.007**
Interpersonal sensitivity	1238	0.43	0.62	0.42	0.43	0.26	1836	0.791	0.4	0.59	1.05	2202	0.293	0.44	0.63	-0.56	3261	0.579
Depression	1237	0.32	0.52	0.28	0.37	1.82	1835	0.069	0.37	0.62	-1.91	2201	0.056	0.4	0.65	-3.51	3260	**0.001**
Anxiety	1237	0.36	0.48	0.34	0.34	0.87	1835	0.387	0.32	0.46	1.92	2201	0.054	0.35	0.48	0.51	3260	0.610
Hostility	1237	0.37	0.45	0.33	0.34	1.75	1835	0.081	0.35	0.44	0.85	2201	0.398	0.4	0.49	-1.98	3260	**0.048**
Phobic anxiety	1238	0.17	0.38	0.15	0.24	1.19	1836	0.233	0.19	0.41	-1.16	2202	0.246	0.19	0.4	-1.39	3261	0.166
Paranoid ideation	1237	0.45	0.57	0.34	0.39	4.31	1835	**<0.001**	0.44	0.57	0.45	2201	0.656	0.48	0.62	-1.34	3260	0.180
Psychoticism	1237	0.24	0.42	0.19	0.28	2.45	1835	**0.014**	0.23	0.48	0.33	2201	0.740	0.25	0.52	-0.78	3260	0.436
GSI	1238	0.35	0.38	0.31	0.23	2.43	1836	**0.015**	0.37	0.43	-1.09	2202	0.277	0.39	0.46	-2.49	3261	**0.013**

S14 Table, contd.

	Swiss general population			German population, Franke 2000					German population, Kliem & Braehler, 2017					German population, Franke, 2017				
	**N**	**Mean**	**SD**	**mean**	**SD**	**t**	**df**	**p**	**mean**	**SD**	**t**	**df**	**p**	**mean**	**SD**	**t**	**df**	**p**
**Males**																		
Somatization	517	0.22	0.33	0.23	0.31	-0.42	815	0.673	0.35	0.46	-5.09	978	**<0.001**	0.34	0.49	-5.00	1527	**<0.001**
Obsessive-compulsive tendencies	517	0.49	0.54	0.5	0.46	-0.23	815	0.815	0.58	0.6	-2.44	978	**0.015**	0.59	0.62	-3.08	1527	**0.002**
Interpersonal sensitivity	517	0.34	0.57	0.35	0.4	-0.34	815	0.733	0.37	0.57	-0.90	978	0.371	0.4	0.57	-2.03	1527	**0.042**
Depression	517	0.30	0.50	0.24	0.32	1.82	815	0.069	0.37	0.64	-1.94	978	0.052	0.41	0.65	-3.40	1527	**0.001**
Anxiety	517	0.31	0.44	0.29	0.31	0.75	815	0.452	0.3	0.42	0.43	978	0.669	0.31	0.43	0.08	1527	0.937
Hostility	517	0.34	0.44	0.29	0.35	1.66	815	0.098	0.36	0.44	-0.75	978	0.453	0.39	0.48	-2.03	1527	**0.043**
Phobic anxiety	517	0.16	0.39	0.14	0.23	0.68	815	0.495	0.18	0.38	-0.95	978	0.343	0.18	0.37	-1.15	1527	0.252
Paranoid ideation	517	0.43	0.57	0.33	0.4	2.82	815	**0.005**	0.48	0.6	-1.21	978	0.226	0.5	0.59	-2.07	1527	**0.039**
Psychoticism	517	0.22	0.41	0.19	0.28	1.18	815	0.237	0.25	0.52	-0.95	978	0.341	0.25	0.49	-1.13	1527	0.258
GSI	517	0.31	0.37	0.28	0.23	1.40	815	0.162	0.37	0.43	-2.21	978	**0.027**	0.38	0.44	-2.95	1527	**0.003**

S14 Table, contd.

	Swiss general population			German population, Franke 2000					German population, Kliem & Braehler, 2017					German population, Franke, 2017				
	**N**	**Mean**	**SD**	**mean**	**SD**	**t**	**df**	**p**	**mean**	**SD**	**t**	**df**	**p**	**mean**	**SD**	**t**	**df**	**p**
**Females**				**mean**	**SD**	**t**	**df**	**p**										
Somatization	720	0.34	0.41						0.38	0.43	-1.662	1221	0.097	0.41	0.5	-3.11	1731	**0.002**
Obsessive-compulsive tendencies	718	0.55	0.60	0.32	0.33	0.72	1018	0.473	0.56	0.58	-0.324	1219	0.746	0.57	0.62	-0.71	1729	0.479
Interpersonal sensitivity	721	0.51	0.65	0.54	0.43	0.23	1016	0.816	0.43	0.6	2.230	1222	**0.026**	0.48	0.67	0.98	1732	0.330
Depression	720	0.35	0.54	0.49	0.45	0.52	1019	0.602	0.36	0.6	-0.420	1221	0.675	0.39	0.65	-1.48	1731	0.139
Anxiety	720	0.40	0.50	0.33	0.4	0.47	1018	0.638	0.34	0.5	2.146	1221	**0.032**	0.38	0.53	0.89	1731	0.373
Hostility	720	0.39	0.45	0.39	0.36	0.39	1018	0.695	0.34	0.44	1.985	1221	**0.047**	0.4	0.51	-0.36	1731	0.718
Phobic anxiety	721	0.18	0.37	0.37	0.33	0.75	1018	0.456	0.21	0.44	-1.162	1222	0.245	0.2	0.43	-0.86	1732	0.390
Paranoid ideation	720	0.47	0.56	0.16	0.25	0.99	1019	0.324	0.39	0.54	2.362	1221	**0.018**	0.46	0.64	0.20	1731	0.845
Psychoticism	720	0.25	0.43	0.34	0.38	3.56	1018	**<0.001**	0.21	0.45	1.585	1221	0.113	0.26	0.55	-0.40	1731	0.687
GSI	721	0.39	0.38	0.19	0.27	2.27	1018	**0.024**	0.37	0.43	0.700	1222	0.484	0.41	0.49	-1.08	1732	0.280

German norms according to Kliem & Braehler, 2017: N total population: 966; N males=463, N females=503
German norms according to Franke, 2017: N total population: 2025; N males=1012, N females=1013
German norms according to Franke, 2000: N total population: 600; N males=300, N females=300
Highlighted in green: $p<0.05$

S15 Table: Comparison of different T-standardizations of the Swiss general population data using paired t-tests

		**Swiss T-Standardization**		**Franke 2000 T-Standardization**		**Kliem & Braehler T-Standardization[a]**		**Franke 2017 T-Standardization[b]**		Comparison Franke 2000 vs Swiss Standardization		Comparison Kliem & Braehler with Swiss Standardization		Comparison Franke 2017 vs Swiss Standardization		Comparison with Kliem & Braehler vs Franke 2000 Standardization		Comparison Franke 2017 vs Franke 2000 standardization		Comparison Franke 2017 vs Kliem & Braehler	
	N	Mean	SD	Mean	SD	Mean	SD	Mean	SD	t	p	t	p	t	p	t	p	t	p	t	p
**Total population**																					
Somatization	1237	50.8	9.0	50.6	9.7					-4.91	**<0.001**										
Obsessive-compulsive tendencies	1235	50.0	9.3	49.5	10.6					-14.36	**<0.001**										
Interpersonal sensitivity	1238	50.6	8.6	49.7	10.3					-15.34	**<0.001**										
Depression	1237	50.3	8.7	50.3	10.0					0.25	0.802										
Anxiety	1237	50.3	9.1	49.9	10.7					-7.56	**<0.001**										
Hostility	1237	50.1	9.1	50.5	10.2					10.42	**<0.001**										
Phobic anxiety	1238	50.6	8.0	50.7	8.8					4.61	**<0.001**										
Paranoid ideation	1237	50.3	9.0	52.2	10.1					48.52	**<0.001**										
Psychoticism	1237	50.5	8.0	51.4	9.3					21.96	**<0.001**										
GSI	1232	50.0	9.8	49.8	12.5					-3.03	**0.003**										

[a] Kliem and Braehler only provide T-scores for males and females separately, but no scores for the overall population

[b] Franke 2017 only provides T-scores for males and females separately, but no scores for the overall population

S15 Table, contd.

		**Swiss T-Standardization**		**Franke 2000 T-Standardization**		**Kliem & Braehler T-Standardization**		**Franke 2017 T-Standardization**		Comparison Franke 2000 vs Swiss Standardization		Comparison Kliem & Braehler with Swiss Standardization		Comparison Franke 2017 vs Swiss Standardization		Comparison with Kliem & Braehler vs Franke 2000 Standardization		Comparison Franke 2017 vs Franke 2000 standardization		Comparison Franke 2017 vs Kliem & Braehler	
	N	Mean	SD	Mean	SD	Mean	SD	Mean	SD	t	p	t	p	t	p	t	p	t	p	t	p
**Males**																					
Somatization	517	50.7	8.6	50.2	9.2	47.8	7.9	48.1	8.1	-14.56	**<0.001**	-75.19	**<0.001**	-98.98	**<0.001**	-35.96	**<0.001**	-41.70	<0.001	-13.92	**<0.001**
Obsessive-compulsive tendencies	517	50.1	9.5	49.5	10.1	48.8	9.1	49.0	8.8	-15.60	**<0.001**	-45.14	**<0.001**	-28.94	**<0.001**	-12.53	**<0.001**	-7.45	<0.001	-13.10	**<0.001**
Interpersonal sensitivity	517	50.3	8.3	49.1	9.7	50.1	8.0	49.4	8.5	-16.77	**<0.001**	-11.89	**<0.001**	-40.98	**<0.001**	11.34	**<0.001**	4.57	<0.001	24.12	**<0.001**
Depression	517	50.5	8.4	51.2	9.5	50.1	7.7	49.5	8.0	10.46	**<0.001**	-9.40	**<0.001**	-28.55	**<0.001**	-10.59	**<0.001**	-18.35	<0.001	24.51	**<0.001**
Anxiety	517	50.1	8.6	49.9	9.8	50.5	8.7	51.1	8.5	-3.57	**<0.001**	14.48	**<0.001**	68.40	**<0.001**	8.38	**<0.001**	16.25	<0.001	-20.05	**<0.001**
Hostility	517	50.5	8.8	51.5	9.4	50.1	9.1	50.3	8.6	25.77	**<0.001**	-18.00	**<0.001**	-10.19	**<0.001**	-39.19	**<0.001**	-25.16	<0.001	-8.83	**<0.001**
Phobic anxiety	517	51.1	7.7	50.4	8.5	50.0	7.5	50.1	7.8	-10.36	**<0.001**	-50.83	**<0.001**	-58.51	**<0.001**	-6.39	**<0.001**	-4.57	<0.001	-8.93	**<0.001**
Paranoid ideation	517	50.2	9.1	52.1	9.8	49.6	8.5	50.5	8.4	41.96	**<0.001**	-17.78	**<0.001**	6.88	**<0.001**	-36.74	**<0.001**	-20.35	<0.001	-40.29	**<0.001**
Psychoticism	517	50.7	8.1	50.8	8.7	50.9	7.1	50.4	7.6	1.68	0.094	3.47	**0.001**	-11.13	**<0.001**	0.56	0.579	-5.69	<0.001	17.56	**<0.001**
GSI	517	50.0	9.7	49.5	11.9	48.8	9.6	49.3	9.1	-4.31	**<0.001**	-32.41	**<0.001**	-16.47	**<0.001**	-5.99	**<0.001**	-1.52	0.129	-15.07	**<0.001**

S15 Table, contd.

		**Swiss T-Standardization**		**Franke 2000 T-Standardization**		**Kliem & Braehler T-Standardization**		**Franke 2017 T-Standardization**		Comparison Franke 2000 vs Swiss Standardization		Comparison Kliem & Braehler with Swiss Standardization		Comparison Franke 2017 vs Swiss Standardization		Comparison with Kliem & Braehler vs Franke 2000 Standardization		Comparison Franke 2017 vs Franke 2000 standardization		Comparison Franke 2017 vs Kliem & Braehler	
	N	Mean	SD	Mean	SD	Mean	SD	Mean	SD	t	p	t	p	t	p	t	p	t	p	t	p
**Females**																					
Somatization	720	50.6	9.1	50.8	10.3	49.1	9.5	48.9	9.2	4.66	**<0.001**	-73.37	**<0.001**	-83.31	**<0.001**	-37.81	**<0.001**	-34.67	**<0.001**	12.02	**<0.001**
Obsessive-compulsive tendencies	718	50.2	9.3	49.5	10.9	49.9	9.5	50.2	9.2	-9.23	**<0.001**	-11.92	**<0.001**	-1.02	0.308	6.21	**<0.001**	9.12	**<0.001**	-13.03	**<0.001**
Interpersonal sensitivity	721	50.1	9.1	49.4	10.9	51.7	9.2	51.4	9.0	-9.81	**<0.001**	62.04	**<0.001**	51.26	**<0.001**	27.56	**<0.001**	22.54	**<0.001**	12.59	**<0.001**
Depression	720	50.3	8.5	49.7	10.1	50.6	8.4	50.3	8.1	-9.93	**<0.001**	8.51	**<0.001**	-0.90	0.366	11.86	**<0.001**	7.08	**<0.001**	17.74	**<0.001**
Anxiety	720	50.0	9.3	49.2	11.0	52.0	9.0	51.5	9.2	-10.35	**<0.001**	70.40	**<0.001**	54.10	**<0.001**	31.09	**<0.001**	23.96	**<0.001**	22.96	**<0.001**
Hostility	720	50.1	8.9	49.7	10.7	51.6	9.2	50.9	9.1	-6.13	**<0.001**	49.76	**<0.001**	31.32	**<0.001**	29.38	**<0.001**	16.44	**<0.001**	33.46	**<0.001**
Phobic anxiety	721	50.7	8.0	50.3	9.4	50.7	7.8	50.8	8.0	-6.96	**<0.001**	-4.02	**<0.001**	0.57	0.568	5.16	**<0.001**	7.10	**<0.001**	-7.55	**<0.001**
Paranoid ideation	720	50.3	8.9	52.2	10.4	52.0	8.9	51.4	8.7	30.33	**<0.001**	48.86	**<0.001**	38.52	**<0.001**	-4.02	**<0.001**	-11.14	**<0.001**	26.00	**<0.001**
Psychoticism	720	50.5	8.0	51.3	10.3	52.0	8.3	51.7	8.0	8.80	**<0.001**	39.42	**<0.001**	39.38	**<0.001**	8.05	**<0.001**	4.32	**<0.001**	16.11	**<0.001**
GSI	715	49.9	9.8	49.5	13.1	50.8	9.8	50.3	9.9	-2.44	**0.015**	25.23	**<0.001**	10.24	**<0.001**	9.36	**<0.001**	5.49	**<0.001**	23.54	**<0.001**
